# Genome-Wide Meta-Analysis of Myopia and Hyperopia Provides Evidence for Replication of 11 Loci

**DOI:** 10.1371/journal.pone.0107110

**Published:** 2014-09-18

**Authors:** Claire L. Simpson, Robert Wojciechowski, Konrad Oexle, Federico Murgia, Laura Portas, Xiaohui Li, Virginie J. M. Verhoeven, Veronique Vitart, Maria Schache, S. Mohsen Hosseini, Pirro G. Hysi, Leslie J. Raffel, Mary Frances Cotch, Emily Chew, Barbara E. K. Klein, Ronald Klein, Tien Yin Wong, Cornelia M. van Duijn, Paul Mitchell, Seang Mei Saw, Maurizio Fossarello, Jie Jin Wang, DCCT/EDIC Research Group, Ozren Polašek, Harry Campbell, Igor Rudan, Ben A. Oostra, André G. Uitterlinden, Albert Hofman, Fernando Rivadeneira, Najaf Amin, Lennart C. Karssen, Johannes R. Vingerling, Angela Döring, Thomas Bettecken, Goran Bencic, Christian Gieger, H.-Erich Wichmann, James F. Wilson, Cristina Venturini, Brian Fleck, Phillippa M. Cumberland, Jugnoo S. Rahi, Chris J. Hammond, Caroline Hayward, Alan F. Wright, Andrew D. Paterson, Paul N. Baird, Caroline C. W. Klaver, Jerome I. Rotter, Mario Pirastu, Thomas Meitinger, Joan E. Bailey-Wilson, Dwight Stambolian

**Affiliations:** 1 National Human Genome Research Institute, National Institutes of Health, Baltimore, Maryland, United States of America; 2 Department of Epidemiology, Johns Hopkins Bloomberg School of Public Health, Baltimore, Maryland, United States of America; 3 Institute of Human Genetics, Technische Universität München, Munich, Germany; 4 Institute of Population Genetics, National Research Council of Italy, Sassari, Italy; 5 Institute for Translational Genomics and Population Sciences, Los Angeles BioMedical Research Institute at Harbor-UCLA Medical Center, Torrance, California, United States of America; 6 Department of Ophthalmology, Erasmus Medical Center, Rotterdam, the Netherlands; 7 Department of Epidemiology, Erasmus Medical Center, Rotterdam, the Netherlands; 8 MRC Human Genetics Unit, IGMM, University of Edinburgh, Edinburgh, United Kingdom; 9 Centre for Eye Research Australia, University of Melbourne, Royal Victorian Eye and Ear Hospital, Melbourne, Australia; 10 Program in Genetics and Genome Biology, The Hospital for Sick Children, Toronto, Ontario, Canada, and DCCT/EDIC Research Group, The Diabetes Control and Complications Trial and Follow-up Study, The Biostatistics Center, The George Washington University, Rockville, Maryland, United States of America; 11 Department of Twin Research & Genetic Epidemiology, King's College London, St Thomas' Hospital, London, United Kingdom; 12 Medical Genetics Institute, Cedars-Sinai Medical Center, Los Angeles, California, United States of America; 13 Division of Epidemiology and Clinical Applications, National Eye Institute, National Institutes of Health, Bethesda, Maryland, United States of America; 14 Department of Ophthalmology and Visual Sciences, University of Wisconsin School of Medicine and Public Health, Madison, Wisconsin, United States of America; 15 Singapore Eye Research Institute, National University of Singapore, Singapore, Singapore; 16 Centre for Vision Research, Department of Ophthalmology and Westmead Millennium Institute, University of Sydney, Sydney, Australia; 17 Department of Epidemiology and Public Health, Yong Loo Lin School of Medicine, National University of Singapore, Singapore, Singapore; 18 Dipartimento di Scienze Chirurgiche, Clinica Oculistica Universita' degli studi di Cagliari, Cagliari, Italy; 19 The Diabetes Control and Complications Trial and Follow-up Study, The Biostatistics Center, The George Washington University, Rockville, Maryland, United States of America; 20 Croatian Centre for Global Health, University of Split Medical School, Split, Croatia; 21 Centre for Population Health Sciences, University of Edinburgh, Edinburgh, United Kingdom; 22 Department of Clinical Genetics, Erasmus Medical Center, Rotterdam, the Netherlands; 23 Department of Internal Medicine, Erasmus Medical Center, Rotterdam, the Netherlands; 24 Netherlands Consortium for Healthy Ageing, Netherlands Genomics Initiative, The Hague, the Netherlands; 25 Institute of Epidemiology, Helmholtz Zentrum München, Neuherberg, Germany; 26 Institute of Human Genetics, Helmholtz Zentrum München, Neuherberg, Germany; 27 Department of Ophthalmology, Hospital “Sestre Milosrdnice”, Zagreb, Croatia; 28 Institute of Genetic Epidemiology, Helmholtz Zentrum München, Neuherberg, Germany; 29 Princess Alexandra Eye Pavilion, Edinburgh, United Kingdom; 30 MRC Centre of Epidemiology for Child Health, Institute of Child Health, University College London, London, United Kingdom; 31 Institute of Ophthalmology, University College London, London, United Kingdom; 32 Ulverscroft Vision Research Group, Institute of Child Health, University College London, London, United Kingdom; 33 Department of Ophthalmology, University of Pennsylvania, Philadelphia, Pennsylvania, United States of America; MOE Key Laboratory of Environment and Health, School of Public Health, Tongji Medical College, Huazhong University of Science and Technology, China

## Abstract

Refractive error (RE) is a complex, multifactorial disorder characterized by a mismatch between the optical power of the eye and its axial length that causes object images to be focused off the retina. The two major subtypes of RE are myopia (nearsightedness) and hyperopia (farsightedness), which represent opposite ends of the distribution of the quantitative measure of spherical refraction. We performed a fixed effects meta-analysis of genome-wide association results of myopia and hyperopia from 9 studies of European-derived populations: AREDS, KORA, FES, OGP-Talana, MESA, RSI, RSII, RSIII and ERF. One genome-wide significant region was observed for myopia, corresponding to a previously identified myopia locus on 8q12 (p = 1.25×10^−8^), which has been reported by Kiefer *et al.* as significantly associated with myopia age at onset and Verhoeven *et al.* as significantly associated to mean spherical-equivalent (MSE) refractive error. We observed two genome-wide significant associations with hyperopia. These regions overlapped with loci on 15q14 (minimum p value = 9.11×10^−11^) and 8q12 (minimum p value 1.82×10^−11^) previously reported for MSE and myopia age at onset. We also used an intermarker linkage- disequilibrium-based method for calculating the effective number of tests in targeted regional replication analyses. We analyzed myopia (which represents the closest phenotype in our data to the one used by Kiefer *et al.*) and showed replication of 10 additional loci associated with myopia previously reported by Kiefer *et al*. This is the first replication of these loci using myopia as the trait under analysis. “Replication-level” association was also seen between hyperopia and 12 of Kiefer *et al.*'s published loci. For the loci that show evidence of association to both myopia and hyperopia, the estimated effect of the risk alleles were in opposite directions for the two traits. This suggests that these loci are important contributors to variation of refractive error across the distribution.

## Introduction

Refractive errors (RE) are etiologically complex, multifactorial disorders characterized by a mismatch between the optical focal length of the eye and its axial length. This optical mismatch causes images to be focused away from the retina. The two major subtypes of spherical RE are myopia (nearsightedness) and hyperopia (farsightedness). Clinically significant myopia affects at least 25% of individuals over age 40 in the United States and western Europe, while hyperopia affects about 10% of individuals in this same age group [1]. Recent reports show that the prevalence of myopia has increased significantly in the United States over the last 3 decades; myopia of 2 (D) diopters or more was estimated to afflict 41.6% of Americans aged 12 to 54 years in 1999–2004, compared to only 25% in 1971–1972 [2]. The myopia epidemic is most acute in East Asia, where prevalence estimates of myopia (of at least 0.5 D) routinely surpass 70% among late teenagers and young adults [3], [4], [5]. A recent study of 19 year-old male military conscripts from Seoul, Korea, found that a staggering 96.5% were myopic [6].

The causes of RE are complex and are a combination of environmental and genetic factors [7]. Twin studies have reported a heritability greater than 0.50 for RE [8]. Several studies have calculated the heritability to be as high as 0.98 for myopia and 0.75 for hyperopia [9], [10], [11], [12]. The search for environmental factors influencing RE have mostly focused on myopia. These include near work and time spent outdoors during childhood and teenage years [13], [14], [15], [16].

Genome-wide association studies have become an essential tool in the study of traits such as RE, and to date there have been 67 published loci for refraction phenotypes [17]. In particular, Kiefer *et al.* [18] performed a genome-wide association study of myopia using self-reported age at onset in 45,771 participants and found 22 significant genome-wide associations. Verhoeven *et al.* [19] performed a genome wide association of the quantitative trait mean spherical equivalent (MSE) and found 24 significant genome-wide associations (2 of which were replications of previously published loci). [19]. Thirteen loci were genome-wide significant in both the Kiefer *et al.* and Verhoeven *et al.* studies [20].

Here we present the results of a genome-wide association meta-analysis of 2 dichotomous RE traits, myopia and hyperopia (adjusted for age, sex and years of education), in 9 populations: the Age-Related Eye Disease Study (AREDS), the Cooperative Health Research in the Region of Augsburg (KORA) the Framingham Eye Study (FES), Ogliastra Genetic Park-Talana (OGP-Talana) Study, the Multi-ethnic Study of Atherosclerosis (MESA), the Rotterdam Eye Studies I, II and III (RSI, RSII, RSIII) and the Erasmus Rucphen Family Study (ERF). These are termed the discovery meta-analyses of myopia and hyperopia hereafter. Eight of the discovery samples were previously included in the meta-analysis of refractive error by Verhoeven *et al.* [19]. One sample, the MESA study, was not included in either Kiefer *et al.* [18]or Verhoeven *et al.*'*s* [19], [21]studies. We attempted replication of significant and suggestive associations from the discovery meta-analyses through meta-analysis of association studies using these same trait definitions to these selected regions in 8 additional studies: the 1958 British Birth Cohort, the Blue Mountains Eye Study (BMES), the CROATIA-Vis Island Study, the CROATIA-Korcula Study, the Diabetes Control and Complications Trial (DCCT), the Orkney Complex Disease Study (ORCADES), the TwinsUK Study, and the Wisconsin Epidemiologic Study of Diabetic Retinopathy (WESDR). All of these studies were previously included in the meta-analysis of refractive error by Verhoeven *et al.* [19]. Finally, we examined the results of our discovery meta-analyses of myopia and hyperopia in the regions found to be associated with myopia age at onset by Kiefer *et al.* [18]. In genetic association studies, the term replication is generally used to mean detection of statistical association of the same trait to the same associated genetic locus in an independent set of data. Here, we also use the term replication when discussing the results of our myopia trait (adjusted for age at examination, sex and years of education) since it is expected to be quite similar to the age at onset of myopia trait used by Kiefer et al. [18] in their study. We show independent replication of 11 of Kiefer et al.'s loci for myopia age at onset [18], and while our myopia trait is not exactly the same as that of Kiefer *et al.* [18], it is the closest phenotype available in our data. We also examined these same regions for association to hyperopia. The association to hyperopia would not constitute a “replication” of Kiefer *et al.*'s myopia findings, but association with this related trait may help to clarify the complex genetic underpinnings of refractive error.

## Materials and Methods

### Populations

The nine GWASs meta-analyzed in the discovery GWAS portion of this study included subjects aged 35–84 years from the Cooperative Health Research in the Region of Augsburg Study (KORA F3, Southern Germany), subjects aged 55–80 from the Age-related Eye Study (AREDS), unrelated subjects aged 28–84 from the Framingham Eye Study (FES), subjects aged 46-86 from the Multi-Ethnic Study of Atherosclerosis (MESA) study, and subjects aged 18–88 from the Ogliastra Genetic Park-Talana (OGP-Talana) study in Sardinia, subjects aged 55 and older from the Rotterdam Eye Study I, subjects aged 55 and older from the Rotterdam Eye Study II, subjects aged 45 and older from the Rotterdam Eye Study III, and subjects aged 18–86 from the ERF study, resulting in a total sample size of 16,830 individuals for the myopia analyses and 14,981 for the hyperopia analyses. All individuals were of European ancestry. This study involved meta-analysis of aggregate statistics from multiple studies. Approval was obtained by the local ethics committees for all studies, all studies were conducted according to the principles expressed in the Declaration of Helsinki and informed consent was obtained from the study participants at all study sites.

### Study design

GWAS analyses of genotype data imputed to HapMap-II were performed for the traits myopia and hyperopia (adjusted for age at examination, sex and years of education) in 9 studies: the Age-Related Eye Disease Study (AREDS), the “Kooperative Gesundheitsforschung in der Region Augsburg” (KORA, “Cooperative Health Research in the Region of Augsburg”), the Framingham Eye Study (FES), the Ogliastra Genetic Park – Talana (OGP-Talana) study, the Multiethnic Study of Atherosclerosis (MESA) and the Rotterdam Eye Studies RSI, RSII, RSIII and the Erasmus Rucphen Family Study (ERF). The results from these analyses were then combined into a discovery meta-analysis GWAS of each trait. Fixed effects meta-analyses were performed with METAL [22] using p values and the effective sample size for each population. METAL calculates a genomic control value [23] for each population and then adjusts each population's results using the corresponding λ value. The discovery meta-analysis genome-wide significance threshold was taken to be 5×10^−8^.

In an attempt to replicate our discovery meta-analysis results and to increase the power of the analyses using our discovery dataset, we obtained association results from 8 other studies, the Blue Mountains Eye Study (BMES), CROATIA-Split, CROATIA-Vis Island, CROATIA-Korcula studies, the Diabetes Control and Complications Trial (DCCT), and the Orkney Complex Disease Study (ORCADES) (Supplemental Methods), just for 30 genomic regions that contained SNPs with association p-values less than 1×10^−5^ to either myopia (11 regions) or hyperopia (14 regions) or both (5 regions) in our discovery meta-analysis (the previously well-replicated association region on chromosome 15q14 was excluded). These studies all performed association of SNPs in these regions with myopia and hyperopia (adjusted for age at examination, sex, years of education when available and up to three principal components when there was significant evidence of population stratification in the data). A replication meta-analysis was performed using the same methods as above on association results in the novel genome-wide significant region for the hyperopia trait in these 8 additional datasets. An additional meta-analysis was then performed in these 30 regions combining results from the discovery datasets and these 8 additional studies. All 8 of these additional datasets were part of the Verhoeven et al. study of mean spherical equivalent. This additional analysis and these datasets are described in Materials S1–S3.

### Quality control of discovery datasets

#### AREDS and KORA

Quality control measures are described elsewhere [24] but in brief: Individuals with chromosome abnormalities and sex discrepancies were removed. Cryptic relatedness was estimated by calculating pairwise identical by descent (IBD) coefficients. For each pair with a kinship coefficient of 0.125 or greater, one member of the pair was dropped based on genotyping rate and trait phenotype, preferring to retain the person with higher genotyping rates and more extreme phenotypes. Population stratification was assessed using principal components. Batch effects and patterns of missingness were eliminated by testing each batch against the others using Fisher's Exact test. As AREDS was a multi-center study, we also tested for differences between collection sites. Samples were dropped for poor performance on the array or a genotyping rate of <98%. SNPs were also removed from a population if its call rate was below 99%, its minor allele frequency was below 0.01, or if its distribution departed significantly from Hardy-Weinberg expectations (p<1×10^−4^) in a single population. We additionally dropped SNPs in both populations where HWE p <1×10^−4^ in 1 population and HWE p <1×10−3 in the other. SNPs were also excluded if they showed more than one genotype inconsistency between HapMap control samples and the consensus genotype in the HapMap database or investigator-provided duplicate samples.

#### Framingham Eye Study

Quality control measures are described elsewhere [24] but in brief: Samples were chosen based on pedigree information and genotyping quality. Samples with a genotypic call rate below 95% were not chosen for analysis. The mean call rate for analyzed samples was 99.2% (SD = 0.4%). The final marker list contained 436,494 high-quality SNPs with a minor-allele frequency> = 0.01, a Mendelian error rate below 2% across all pedigrees, a genotype call rate above 95%, and whose distribution was consistent with Hardy-Weinberg expectations (P>1×10^−4^).

#### MESA

For the MESA dataset, SNPs with MAF less than 0.02 or HWE p value less than 0.001 were removed from the analysis. Genotyping was performed using the Affymetrix Genome-Wide Human SNP Array 6.0. IMPUTE version 2.1.0 was used to perform imputation for the MESA Caucasian participants (chromosomes 1–22) using HapMap Phase I and II - CEU as the reference panel (release #24 - NCBI Build 36 (dbSNP b126)). SNPs with genotype call rate less than 0.95, MAF less than 0.02, HWE p value less than 0.001, or oevar less than 0.3 were removed from the analysis. Association tests were performed by SNPTEST v2 (Marchini et al., 2007).

#### OGP-Talana

Quality control of the SNP data was performed using the GenABEL software package in R. Samples with overall SNP call rate <93%, with minor allele frequency <0.01, with Hardy-Weinberg P value>10^−6^, showing excess heterozygosity, or being classified as outliers by allelic identity-by-state (IBS) clustering analysis, were excluded.

### Rotterdam eye studies I,II and III

Subjects with cataracts and history of cataract or refractive surgery were excluded from the study. DNA was extracted from blood leucocytes according to standard procedures. Genotyping of SNPs was performed using the Illumina Infinium II HumanHap550 chip v3.0 array (RS-I); the HumanHap550 Duo Arrays and the Illumina Human610-Quad Arrays (RS-II), and the Illumina Human 610 Quad Arrays (RS-III). Samples with low call rate (<97.5%), with excess autosomal heterozygosity (>0.336), or with sex-mismatch were excluded, as were outliers identified by the identity-by-state clustering analysis (outliers were defined as being>3 s.d. from population mean or having identity-by-state probabilities>97%). GWAS analyses were performed using GRIMP.

### Erasmus rucphen family study

Subjects with cataracts and history of cataract or refractive surgery were excluded from the study. DNA was genotyped on one of four different platforms (Illumina 6k, Illumina 318K, Illumina 370K and Affymetrix 250K). Samples with low call rate (<97.5%), with excess autosomal heterozygosity (>0.336), or with sex-mismatch were excluded, as were outliers identified by the identity-by-state clustering analysis (outliers were defined as being>3 s.d. from population mean or having identity-by-state probabilities>97%). GWAS analyses were performed using the ProbABEL package from the ABEL set. A lambda correction was performed to adjust for cryptic relationship.

### Genotype imputation of data

To produce a consensus set of genotypes for imputing to the HapMap-II, AREDS and KORA high quality SNPs were filtered to those present on HapMap-II. Imputation to the HapMap-II reference panel (CEU population release 22, NCBI build 36) was performed in MACH [22], [25] in 2 stages. Stage one was the model parameter estimation stage which used a random sample of 300 individuals from each population, using the greedy option which only uses the reference haplotypes (supplied here from the HapMap) and 100 Markov Chain iterations. Stage two is the actual imputation stage and uses the model parameters estimated in stage one to speed up the imputation of the genotypes. After imputation, the remaining high quality genotyped SNPs were merged back in with the SNPs from the imputation procedure for the AREDS and KORA data. For the FES data, genotype imputation to the HapMap-II reference panel (CEU population release 22, NCBI build 36) was carried out in a two-step process using the Markov Chain Haplotyping (MACH version 1.0.16.a) software. First, crossover and error-rate maps were built using 400 unrelated individuals (200 male and 200 female) sampled from FHS subjects. Second, genotype imputations of approximately 2.5 million autosomal HapMap-II SNPs were carried out on the entire FHS dataset using parameters estimated from step 1. For MESA, IMPUTE version 2.1.0 was used to perform imputation for the Caucasian participants (chromosomes 1-22) using HapMap Phase I and II - CEU as the reference panel (release #24 - NCBI Build 36 (dbSNP b126)). For OGP-Talana, using the phase II CEU HapMap individuals (release 22, NCBI build 36) as reference panel for imputation, genotypes were imputed for nearly 2.5 million SNPs using MACH. SNPs imputed with Rsq <0.3 were excluded. For RSI,II and III and ERF, a set of genotyped input SNPs with call rate>98%, with minor allele frequency>0.01, and with Hardy-Weinberg P value>10^−6^ was used for imputation. We used the Markov Chain Haplotyping (MACH) package version 1.0.15 software (Rotterdam, The Netherlands; imputed to plus strand of NCBI build 36, HapMap release #22) for the analyses. For each imputed SNP, a reliability of imputation was estimated as the ratio of the empirically observed dosage variance to the expected binomial dosage variance (O/E ratio).

### Data analysis

Genetic association was estimated by fitting a logistic regression model separately to the traits myopia and hyperopia. To create the dichotomous traits, we calculated mean spherical equivalent (MSE) as the average of spherical equivalent (SE) of refraction between the two eyes, or the single SE value for persons with only a single SE measurement. For myopia, cases were defined as MSE <−1D, controls>0D and individuals between 0D and −1D coded as unknown. For hyperopia, cases were defined as MSE>+1D, controls <0D and individuals between 0D and +1D coded as unknown. A general additive genetic model was used to code the SNP effect (i.e. SNPs were coded according to the number of minor alleles [0,1,2] for each person); covariates included age; sex; and years of education. For AREDS, KORA and FES, this was accomplished using the PLINK (version 1.07) statistical software (http://pngu.mgh.harvard.edu/~purcell/plink) [26]. For AREDS analyses, the first three principal components (eigenvectors) of the EIGENSTRAT analysis were also included along with the covariates listed above. For MESA, these association tests were performed by SNPTEST v2.52. For OGP-Talana, all regression models were run using the ProbABEL package from the ABEL set of programs which adjusts jointly for cryptic relationship and population stratification. For RSI, II and III and ERF, we used genomic control [23] to obtain optimal and unbiased results and applied the inverse variance method of each effect size estimated for both autosomal SNPs that were genotyped and imputed in both cohorts.

Association analyses were performed for both traits and a genome-wide meta-analysis was performed on the 9 populations and 8 replication data sets (Blue Mountains Eye Study, Croatia Vis Island Study, Croatia Korcula Study, Diabetes Control and Complications Trial, Orkney Complex Disease Study, UK Twins Study, 1958 British Birth Cohort, Wisconsin Epidemiologic Study of Diabetic Retinopathy). Details of the genome-wide analyses of the individual discovery datasets and the replication analyses are shown in the supplemental methods and results including QQ-plots and Manhattan plots for each of the discovery cohorts in Figures S1-S9. Figure S10 is a flowchart showing the workflow of the entire study.

### SNP selection for replication

Thirty genomic regions that contained SNPs with association p-values less than 1×10^−5^ to either myopia (11) or hyperopia (14) or both (5) in our discovery meta-analysis (excluding the 15q14 region) were chosen for replication or further study in the 8 additional datasets. We analyzed all SNPs within a 500 kb window centered on the most significant SNP in each region from the discovery meta-analysis.

For the comparison of our discovery meta-analysis results with the myopia age at onset loci from the Kiefer *et al.* [18] study, a list of strongly associated variants that were genome-wide significant (p≤5×10^−8^) or suggestive (p<1×10^−6^) in Kiefer *et al.* [18] was selected. We analyzed all SNPs within a 500 kb window centered on these replication SNPs in our data.

### Calculation of effective number of tests and replication significance thresholds

It has become increasingly clear that only attempting to replicate the exact SNPs found to be genome-wide significant in a discovery GWAS can produce a failure to replicate due to underlying differences in linkage disequilibrium (LD) and allele frequencies [27], [28], even in populations self-identified as having the same ethnicity. Ioannidis *et al.* [29] have shown that restricting replication efforts to only a few of the most significant SNPs from an associated region leads to less robust information for those loci. The resulting failure to replicate may be because those selected SNP(s) are not necessarily more informative or closer to the causal variant than other SNPs in the region. Several approaches to this problem have been proposed, including incorporating linkage information [30], pathway-based association [31] and other methods which use multiple SNPs in the analysis [32], [33], [34], [35], [36], [37], [38], [39]. A linkage disequilibrium (LD) based binning strategy, proposed by Christoferou [39] may prove to be the most useful. However, the issues of handling SNPs which map to more than one gene due to overlapping reading frames and the correlations between genes and derivative gene scores still need to be resolved. Until that problem has a solution, it may be more powerful to study a dense panel of SNPs from each associated region, and utilize imputation to the latest version of 1000 Genomes data to provide additional genotypes to harmonize available SNPs across studies even when genotyped on different platforms. Here we selected all SNPs that were within a specified window of the original SNP and used the method of Ramos et al. [40] to model the LD structure in one of the replication populations to calculate the effective number of independent tests being performed across all of our replication regions. Traditional methods of correcting for multiple comparisons, such as the widely used Bonferroni correction considering all SNPs tested, are notoriously conservative because they do not take intermarker correlation fully into account but treat all the tests as independent. By using the effective number of independent tests in a Bonferroni correction, Type I error is still controlled and power is improved. Various approaches to calculating the effective number of independent tests when using such a regional replication strategy have been proposed since many of the SNPs in such a region are in LD with each other and do not represent independent tests [41], [42], [43], [44], [45], although many of these approaches are still overly conservative. The Ramos et al. [40] approach properly accounts for SNP interdependence, allows computation of the effective number of independent tests for very large numbers of highly correlated SNPs and is less computationally intensive than permutation-based methods. We used the method of Ramos *et al.* [40] to calculate the number of effective tests (*N_eff_*) in all the replication regions and divided α by this effective number of tests to calculate the significance threshold separately in the AREDS, KORA and Framingham datasets. The Ramos method calculates *N_eff_* by first estimating the KxK covariance matrix for the K SNPs in the replication regions using the genotype data. Then the covariance matrix is spectrally decomposed to calculate the eigenvalues. The effective number of tests is then estimated using the relationship
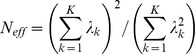
in which λ_k_ is the kth eigenvalue of the K×K covariance matrix for the K SNPs [46]. The Bonferroni-corrected significance threshold is then calculated as α/N*_eff_*.

The markers in each region are very densely spaced, with high levels of LD between markers in each block. The calculations from the AREDS data gave the largest effective number of tests and thus the most conservative Bonferroni-corrected significance threshold; thus this was chosen as our significance threshold for our replication studies. However, the Bonferroni-corrected thresholds derived by applying this method to the KORA and Framingham data were only slightly less conservative than the threshold derived from the AREDS data.

## Results

After all quality control measures and appropriate association analyses, genome-wide association results from Caucasian participants in the AREDS, KORA, FES, OGP-Talana, MESA, RSI, RSII, RSIII and ERF studies were combined in a genome-wide discovery meta-analysis totaling 16,830 individuals for myopia and 14,981 individuals for hyperopia. Table 1 describes the characteristics of the populations after classifying participants into myopia, hyperopia, control or unknown categories.

**Table 1:** Baseline Characteristics of the nine populations.

	AREDS	KORA	FES	MESA	OGP-TALANA	RS1	RS2	RS3	ERF	Total
N	1877	1869	1389	1462	683	5238	2009	1970	2028	18525
Mean Age (SD)	68.0 (4.7)	55.6 (11.8)	55.6 (8.9)	61.9 (9.4)	42.2 (19.1)	68.5 (8.6)	64.2 (7.4)	60.8 (5.5)	48.5 (14.3)	
N Myopia^1^ Cases	346	550	348	486	71	763	395	594	370	3923
Myopia Cases MSE^2^	−2.81	−2.72	−3.08	−3.20	−4.41	−3.21	−3.08	−3.22	−3.03	
N Myopia Controls	1333	840	773	731	428	3964	1374	1056	1197	11696
Myopia Controls MSE^2^	1.59	1.38	1.60	1.70	1.38	1.88	1.72	1.39	1.24	
N Myopia Unknown	198	479	268	245	184	601	240	320	461	
N Hyperopia^3^ Cases	854	424	426	506	64	2779	919	556	540	7068
Hyperopia Cases MSE^2^	2.56	2.30	2.31	2.21	2.42	2.48	2.33	2.23	2.29	
N Hyperopia Controls	600	1010	654	714	153	1350	627	907	829	6844
Hyperopia Controls MSE^2^	−1.76	−1.79	−1.92	−2.32	−2.56	−2.00	−2.09	−2.21	−1.52	
N Hyperopia Unknown	423	435	309	242	466	1109	463	507	659	
Sex (% Male) (Myopia/Hyperopia)	41/40	50/49	42/41	54/43	41/40	48/39	49/44	46/43	53/62	
Myopia λ	1.001	0.997	1.004	1.024	1.085	1.020	1.018	1.015	1.323	1.038
Hyperopia λ	1.020	1.023	0.997	1.017	1.156	1.041	1.024	1.010	1.254	1.046

1. For myopia, cases were defined as MSE <−1D, controls>0D and individuals between 0D and −1D coded as unknown.

2. Average MSE of all cases or controls used in the analyses.

3. For hyperopia, cases were defined as MSE>+1D, controls <0D and individuals between 0D and +1D coded as unknown.

**Figure 1:**
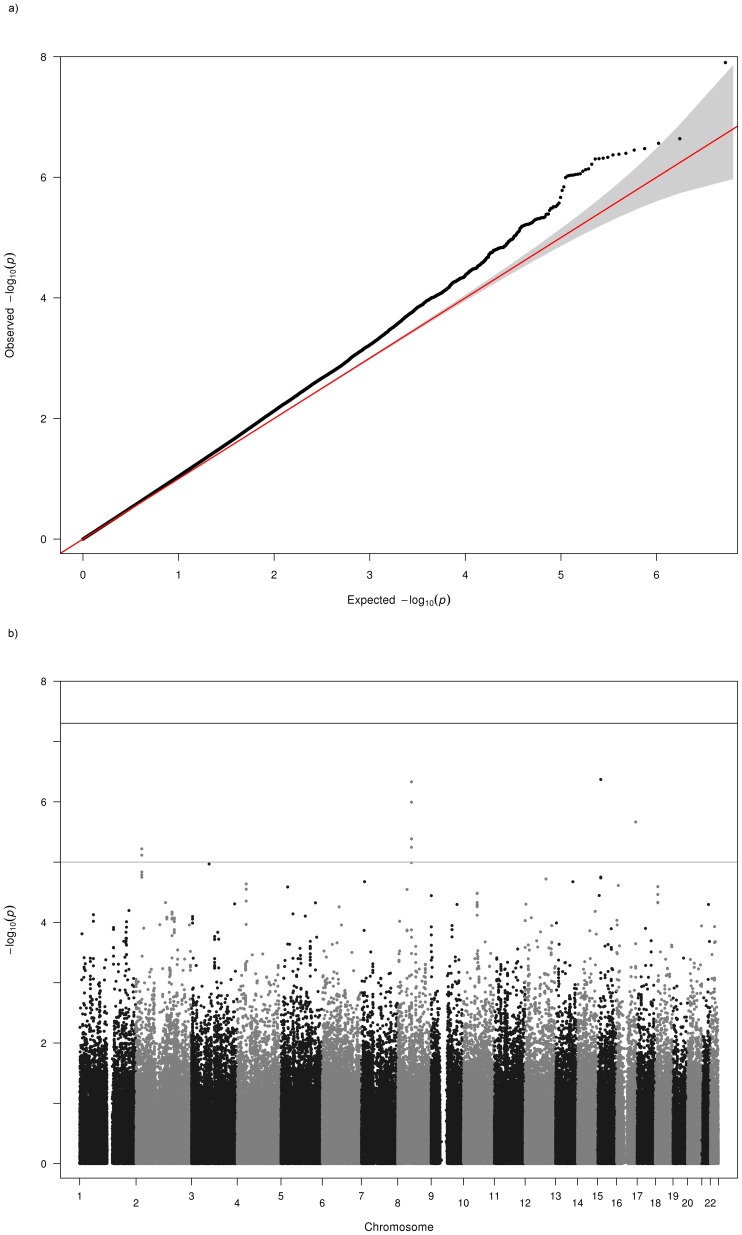
Q-Q and Manhattan Plots for the myopia analysis of all cohorts. a) Q-Q plot for association between all SNPs analyzed and myopia in the meta-analysis. Each dot represents an observed statistic (defined as -log10 P) versus the corresponding expected statistic. The red line corresponds to the null distribution. b) Manhattan plot for association between all SNPs analyzed and myopia in the meta-analysis. Each dot represents an observed statistic (defined as -log10 P). The darker gray line corresponds to the genome-wide significance threshold and the lighter gray line represents the suggestive threshold.

**Figure 2:**
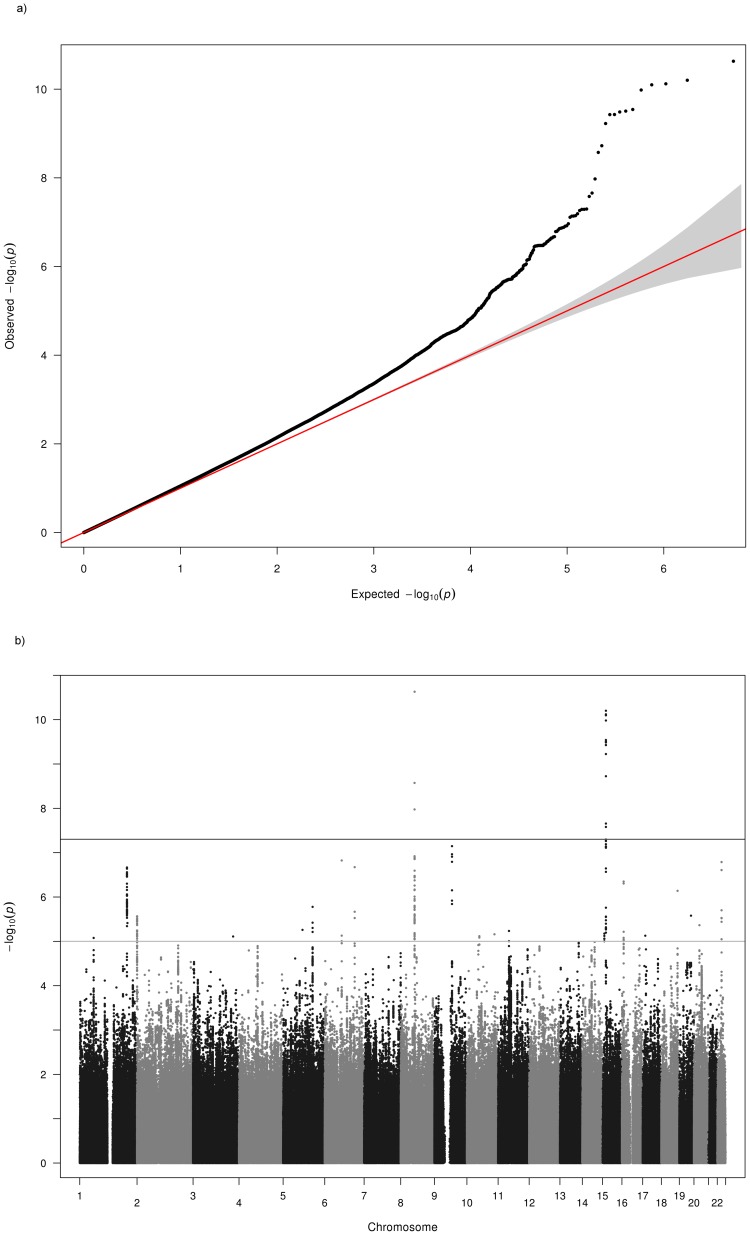
Q-Q and Manhattan Plots for the hyperopia analysis of all cohorts. a) Q-Q plot for association between all SNPs analyzed and hyperopia in the meta-analysis. Each dot represents an observed statistic (defined as -log10 P) versus the corresponding expected statistic. The red line corresponds to the null distribution. b) Manhattan plot for association between all SNPs analyzed and hyperopia in the meta-analysis. Each dot represents an observed statistic (defined as -log10 P). The darker gray line corresponds to the genome-wide significance threshold and the lighter gray line represents the suggestive threshold.

Testing for population stratification using EIGENSOFT and principal components analysis found no evidence of population stratification in KORA, but some evidence of substructure was detected in the AREDS, FES and MESA studies. These were adjusted for in the genome-wide association analyses by including the first three principal components from the PCA as covariates in our regression models. The OGP-Talana data were also adjusted for cryptic relatedness using the ProbABEL R package. For ERF and RS1–3, the population was assumed to be homogeneous and outliers excluded. Genomic control [23] values (λ) calculated by METAL [47] for each population prior to meta-analysis for each trait are given in Table 1. These values were used by METAL to adjust each population's results before including in the fixed effects meta-analysis. The QQ plots of the meta-analysis p values (Figure 1a and Figure 2a) showed some deviation from the null. However, the genomic control method [23] was used to further control for population stratification and inter-population differences in the final meta-analysis. The variance inflation factors calculated by METAL [47] for the final meta-analysis across the nine cohorts for myopia and hyperopia were 1.038 and 1.046 respectively. Lambda values ranging from approximately 0.95 to 1.1 are considered desirable.

Results of the genome-wide meta-analyses are shown in Figure 1b and Figure 2b and results for each sample separately are given in Figure S1 (AREDS), Figure S2 (KORA), Figure S3 (FES), Figure S4 (MESA), Figure S5 (OGPT), Figure S6 (RS-I), Figure S7 (RS-II), Figure S8 (RS-III), Figure S9 (ERF). Eight additional studies (1958 British Birth Cohort, BMES, CROATIA-Vis, CROATIA-Korcula, DCCT, ORCADES, TwinsUK and WESDR) were used for replication and baseline characteristics of these studies can be found in Table S3. Results of further meta-analyses of genomic regions that exhibited suggestive evidence of association with myopia or hyperopia using regional results from the 8 additional studies listed above are given in Tables S6 and S7. Meta-analyses combining the replication region association results from the 9 discovery datasets and the 8 replication datasets did not result in genome-wide significant results, except for the 8q12 locus (results not shown) that was already genome-wide significant in the discovery dataset.

To determine if our discovery meta-analyses showed evidence of association in any of 35 loci (Table S1) reported to exhibit genome-wide significant or suggestive (p<1×10^−6^) association with myopia age at onset by Kiefer *et al.* [18], a total of 33,591 SNPs overlapping all associated loci were selected (Table S2). These included the most significant discovery SNP plus all available genotyped and imputed SNPs within 500kb of the most significant discovery SNP (Table S2). Accounting for all the LD in each region reduced the effective number of tests, *N_eff_*, to 475.71. The replication significance threshold, calculated while taking into account this LD structure in replication regions [40], was 
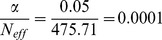
.

### Myopia

Results of the discovery meta-analysis (Figure 1, Table S4) shows one genome-wide significant marker corresponding to a previously identified myopia age at onset [18] and refractive error [19] locus on 8q12 (rs10113215, p = 1.25×10^−8^). We also observed association to the well-replicated locus on 15q14 (near *GJD2*) that was close to genome-wide significant (rs1370156, p = 2.29×10^−7^). No attempt was made to replicate the chromosome 15q14 region since it has been well replicated. SNPs in the 8q12 replication region did not reach the replication threshold (for rs10113215, replication p = 0.02; top replication p-value in the region was p = 0.0022 for rs6995115). For the discovery meta-analysis suggestive regions, one of the selected SNPs achieved the replication threshold for myopia (rs4326350 on 8p23, p = 6.1×10^−5^). However, it should be remembered that this region did not exhibit genome-wide significant association in the discovery meta-analysis (replication p-values in Table S6).

In addition to the 8q12 locus, 10 other myopia age at onset regions from the Kiefer *et al.* study [18] showed significant evidence of replication in our discovery meta-analysis (Table 2). Eight of these loci have also been reported as associated with MSE by Verhoeven *et al.* [19]. However, two of the regions we replicated were not reported significantly associated with MSE by Verhoeven *et al.* [19]. On chromosome 3p26, rs2587916 reached the replication threshold in our discovery meta-analysis (p = 2.79×10^−5^). This SNP is 256 bp away from the SNP reported in this region by Kiefer *et al.* [18], rs1843303 (which had p = 6.32×10^−4^ in our data, Table 2). These two SNPs exhibit strong linkage disequilibrium with an R^2^ of 0.963 and a D′ of 1 in our data. The most significant SNP at the second locus on chromosome 6 is the same SNP as reported by Kiefer *et al.* [18], rs7744813 (p = 6.07×10^−6^, Table 2).

**Table 2:** Results of the replication of regions significantly associated with myopia age at onset by Kiefer *et al.* [18] showing meta-analysis association results for each chosen SNP with myopia in our data.

Replication SNP^1^	Chromosome	Position	Replication P value^2^	Best SNP^3,6^	Offset^4,6^	P value^5,6^	Nearest Gene(s)^7^	Reported by Verhoeven et al.
rs6702767	1	200844547	1.12E-01	rs4471299	391129	1.92E-04		No
rs11681122	2	146786063	N/A	rs10928276	661	4.61E-04		No
rs17428076	2	172851936	7.13E-02	rs3821093	157350	7.50E-03		No
rs1898585	2	178660450	N/A	rs1405645	192929	1.47E-03		No
**rs1550094**	**2**	**233385396**	**N/A**	**rs1656404**	**5456**	**3.72E-05**	***PRSS56***	Yes
**rs1843303**	**3**	**4185124**	**6.32E-04**	**rs2587916**	**256**	**2.79E-05**	***SUMF1/SETMAR***	No
rs7624084	3	141093285	2.93E-02	rs1007118	247701	3.53E-03		No
rs1031004	4	80516849	N/A	rs1440853	10203	4.09E-04		No
**rs5022942**	**4**	**81959966**	**N/A**	**rs1353387**	**12783**	**6.16E-05**	***BMP3***	Yes
**rs7744813**	**6**	**73643289**	**6.07E-06**				***KCNQ5***	No
**rs12193446**	**6**	**129820038**	**8.74E-06**				***LAMA2***	Yes
rs9365619	6	164251746	5.26E-01	rs6900149	211224	2.34E-02		No
**rs2137277**	**8**	**40734662**	**2.84E-05**	**rs4736884**	**5031**	**1.78E-05**	***ZMAT4***	Yes
**chr8:60178580**	**8**	**60178580**	N/A	**rs10113215**	**46386**	**1.25E-08**	***TOX***	Yes
rs10963578	9	18338649	N/A	rs10115405	17893	8.99E-04		No
rs11145746	9	71834380	1.12E-02	rs3002374	35408	2.88E-04		No
**rs4245599**	**10**	**60365755**	**5.75E-05**	**rs12264028**	**87616**	**2.57E-05**	***BICC1***	Yes
rs6480859	10	79081948	5.36E-02	rs16933964	457642	1.00E-03		No
rs745480	10	85986554	6.88E-03	rs4244950	34147	2.12E-04		No
rs4367880	10	114795256	N/A	rs7071843	316234	1.11E-03		No
rs11602008	11	40149305	N/A	rs7924805	61948	1.02E-03		No
chr11:65348347	11	65348347	N/A	rs610037	198510	5.94E-03		No
rs10736767	11	84637065	6.61E-02	rs1940124	18791	6.49E-04		No
rs6487748	12	9435768	N/A	rs12822596	125774	1.83E-03		No
rs3138142	12	56115585	6.68E-02	rs2291615	219566	3.18E-03		No
**rs4291789**	**13**	**100672921**	**N/A**	**rs8000506**	**3929**	**2.98E-05**	***ZIC2/ZIC5***	Yes
rs61988414	14	42313443	N/A	rs12878452	2013	1.61E-03		No
chr14:54413001	14	54413001	N/A	rs12147340	493078	1.43E-03		No
**rs524952**	**15**	**35005886**	**8.74E-05**	**rs1370156**	**21004**	**2.29E-07**	***GJD2***	Yes
rs4778882	15	79382019	N/A	rs925114	323501	6.84E-04		No
**rs17648524**	**16**	**7459683**	**3.03E-06**	**rs4581716**	**1549**	**1.65E-06**	***RBFOX1***	Yes
rs2908972	17	11407259	4.10E-03	rs4792105	295899	1.79E-03		No
rs10512441	17	31239645	2.47E-03	rs17780981	120609	5.52E-04		No
rs9902755	17	47220726	1.51E-01	rs7222737	31323	2.16E-03		No
chr17:79585492	17	79585492	N/A	rs11651296	232337	8.53E-03		No

1. SNPs which are either genome-wide significant or meet our replication threshold are highlighted in bold text. Allele frequencies for these SNPs in each of our discovery populations can be found in Table S8.

2. For each SNP reported by Kiefer *et al.*, Replication P value is the P value of that SNP in our analysis. If that SNP was not genotyped or imputed in our data, it is indicated with N/A.

3. For regions where the most significant SNP in our analysis is not the original reported SNP, that SNP is reported as Best SNP.

4. Offset is the absolute distance in base pairs to the original SNP and the P value associated with Best SNP.

5. Z scores and direction of effect for all SNPs are in Table S2.

6. This column left blank where the original SNP is the most significant SNP in the region.

7. Nearest Gene(s) indicates the closest gene by physical position for these SNPs.

**Figure 3:**
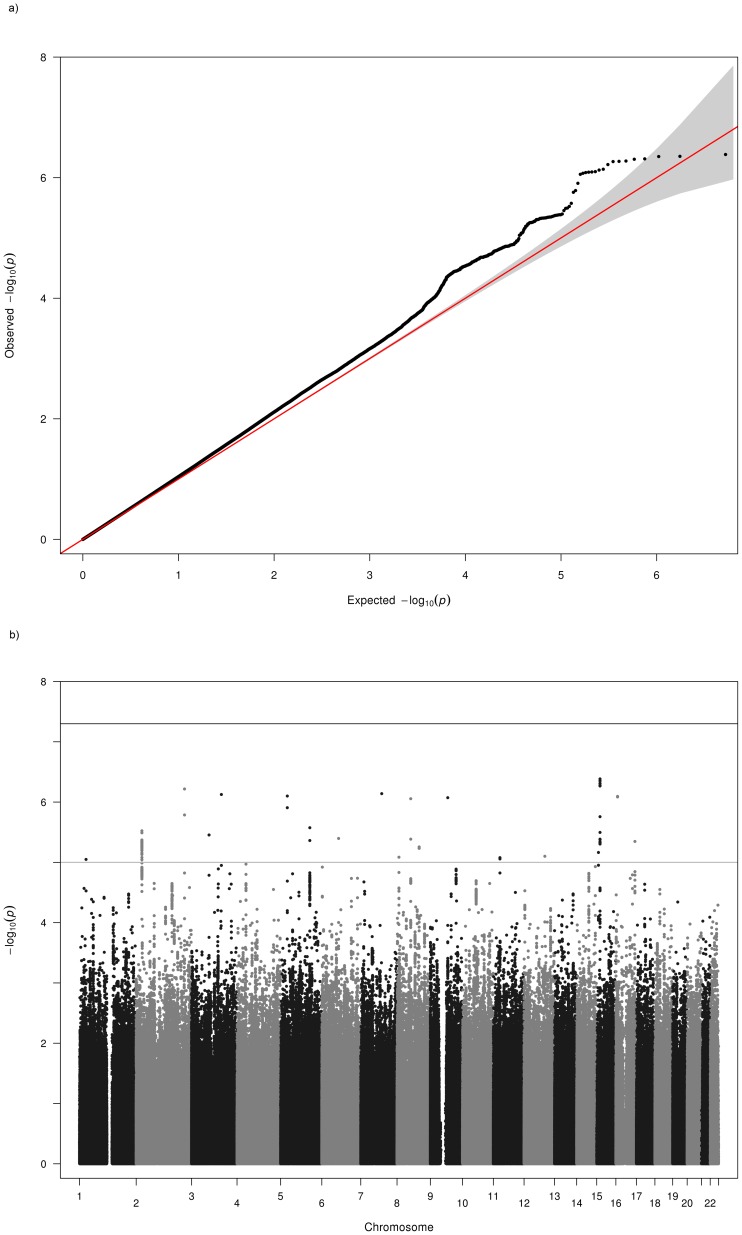
Q-Q and Manhattan Plots for the myopia analysis excluding the ERF cohort a) Q–Q plot for association between all SNPs analyzed and myopia in the meta-analysis excluding the ERF cohort. Each dot represents an observed statistic (defined as -log10 P) versus the corresponding expected statistic. The red line corresponds to the null distribution. b) Manhattan plot for association between all SNPs analyzed and myopia in the meta-analysis excluding the ERF cohort. Each dot represents an observed statistic (defined as -log10 P). The darker gray line corresponds to the genome-wide significance threshold and the lighter gray line represents the suggestive threshold.

Due to the high genomic control values for OGP-Talana and ERF (Table 1), we examined QQ plots of only the common SNPs (MAF>0.2) to see if this made an improvement, since all the associated SNPs reported here have high MAFs. In OGP-Talana this improved the QQ plots (Figure S9) but it made no difference for ERF. Therefore, we dropped ERF from the analysis and re-examined the results (Figure 3). For most loci this made minimal difference to the p values. However, for 3 loci there was a considerable difference. The genome-wide significant result for myopia on chromosome 8 was no longer genome-wide significant (p = 8.8×10^−7^), although it still remained well below our replication significance threshold. The loci on 2q37 and 3p26 were no longer below our replication threshold.

### Hyperopia

Meta-analysis results showed two genome-wide significant associations with hyperopia (Figure 2, Table S5). These regions overlapped with loci on 15q14 (rs11073060, p = 9.11×10^−11^) and 8q12 (rs10089517,p = 1.82×10^−11^) previously reported for MSE in Verhoeven *et al.* [19] and for myopia age at onset in Kiefer *et al.* [18]. No attempt was made to replicate the 15q14 locus since it has been well replicated for MSE. None of the SNPs selected to attempt replication of the discovery meta-analysis genome-wide significant association with hyperopia on chromosome 8q12 achieved the replication threshold (rs10089517, p = 0.08; top replication p-value in the region was 0.014 at rs11778476) (Table S7). In addition, for the discovery meta-analysis suggestive regions, one SNP achieved the replication threshold for hyperopia (rs12660628 on 6q21, p = 7.7×10^−5^). However, it should be remembered that this region did not exhibit genome-wide significant association in the discovery meta-analysis (replication p-values in Table S7).

In addition to the 15q14 and 8q12 loci, 10 other regions (Table 3) that were genome-wide significant in the Kiefer *et al.* [18] analysis of myopia age at onset exhibited p values for association with hyperopia that met our “replication” threshold for these regions. Given this is a different but related trait, this finding is interesting. Five of these regions have been replicated using myopia as the trait in our data here (three of which were also found to be significantly associated with MSE by Verhoeven *et al.* [19]). Verhoeven *et al.* [19] also found that 1 more of these 10 regions (Table 3) showed significant association with MSE. Of the remaining 4 regions from Table 3 the most significant of these 4 SNPs was rs1371993 (p = 1.13×10^−5^), a SNP on chromosome 4, 35Kb from the SNP reported by Kiefer *et al.* [18] for myopia age at onset (rs1031004, not available in our data).

**Table 3:** Results of the hyperopia analyses in the regions that were significantly associated with myopia age at onset by Kiefer *et al.* [18] showing meta-analysis association results for each chosen SNP.

Replication SNP^1^	Chromosome	Position	Replication P value^2^	Best SNP^3,6^	Offset^4,6^	P value^5,6^	Nearest Gene(s)^7^	Reported by Verhoeven et al.
rs6702767	1	200844547	1.60E-01	rs6703834	264384	4.58E-03		No
rs11681122	2	146786063	N/A	rs17412774	12116	1.50E-04		No
rs17428076	2	172851936	6.43E-03	rs3821093	157350	2.44E-04		No
**rs1898585**	**2**	**178660450**	**N/A**	**rs6718702**	**84399**	**1.47E-05**	***PDE11A***	No
**rs1550094**	**2**	**233385396**	**N/A**	**rs1881494**	**12631**	**4.63E-05**	***PRSS56***	Yes
**rs1843303**	3	4185124	1.98E-05	rs795294	826	1.18E-05	*SUMF1/SETMAR*	No
rs7624084	3	141093285	N/A	rs9821337	2901	1.88E-04		No
**rs1031004**	**4**	**80516849**	**N/A**	**rs1371993**	**35034**	**1.13E-05**	***GK2*** ** (MIM:137028)**	No
rs5022942	4	81959966	N/A	rs2201544	30290	4.94E-03		Yes
**rs7744813**	**6**	**73643289**	**7.00E-08**				***KCNQ5***	No
**rs12193446**	**6**	**129820038**	**1.84E-07**				***LAMA2***	Yes
rs9365619	6	164251746	2.67E-01	rs2759387	412079	9.50E-03		No
rs2137277	8	40734662	2.72E-02	rs6474290	94596	2.42E-03		Yes
**chr8:60178580**	**8**	**60178580**	**N/A**	**rs10089517**	**141**	**1.82E-11**	***TOX***	Yes
rs10963578	9	18338649	N/A	rs10115405	17893	2.54E-04		No
rs11145746	9	71834380	8.33E-03	rs10481782	22378	2.71E-04		No
rs4245599	10	60365755	1.16E-03	rs1866168	4194	8.11E-04		Yes
rs6480859	10	79081948	1.45E-02	rs16933964	457642	4.35E-04		No
rs745480	10	85986554	3.26E-01	rs17103281	25190	1.06E-04		No
rs4367880	10	114795256	N/A	rs7914029	215000	3.40E-04		No
**rs11602008**	**11**	**40149305**	**N/A**	**rs10837366**	**75045**	**7.61E-05**	***LRRC4C*** ** (MIM:608817)**	No
chr11:65348347	11	65348347	N/A	rs11820062	81589	7.56E-03		No
rs10736767	11	84637065	1.99E-01	rs10898278	303825	3.05E-03		No
rs6487748	12	9435768	N/A	rs7305636	157088	9.29E-04		No
rs3138142	12	56115585	4.32E-02	rs12828230	230568	5.87E-04		No
**rs4291789**	**13**	**100672921**	**N/A**	**rs1347190**	**24823**	**6.65E-06**	***ZIC2/ZIC5***	Yes
rs61988414	14	42313443	N/A	rs10149831	125528	1.35E-03		No
chr14:54413001	14	54413001	N/A	rs17127526	444960	1.26E-03		No
**rs524952**	**15**	**35005886**	**3.07E-08**	**rs11073060**	**16036**	**9.11E-11**	***GJD2***	Yes
rs4778882	15	79382019	N/A	rs1443658	4348	2.88E-03		No
**rs17648524**	**16**	**7459683**	**4.86E-07**				***RBFOX1***	Yes
rs2908972	17	11407259	1.39E-04	rs12602611	166838	1.26E-05	*SHISA6*	No
rs10512441	17	31239645	4.78E-03	rs17183113	210521	2.40E-03		No
rs9902755	17	47220726	2.81E-01	rs8064938	439898	1.73E-03		No
chr17:79585492	17	79585492	N/A	rs6565596	60374	1.13E-02		No

1. SNPs which are either genome-wide significant or meet our replication threshold are highlighted in bold text. Allele frequencies for these SNPs in each of our discovery populations can be found in Table S8.

2. For each SNP reported by Kiefer *et al. *, Replication P value is the P value of that SNP in our analysis. If that SNP was not genotyped or imputed in our data, it is indicated with N/A.

3. For regions where the most significant SNP in our analysis is not the original reported SNP, that SNP is reported as Best SNP.

4. Offset is the absolute distance in base pairs to the original SNP and the P value associated with Best SNP.

5. Z scores and direction of effect for all SNPs are in Table S2.

6. This column left blank where the original SNP is the most significant SNP in the region.

7. Nearest Gene(s) indicates the closest gene by physical position for these SNPs.

**Figure 4:**
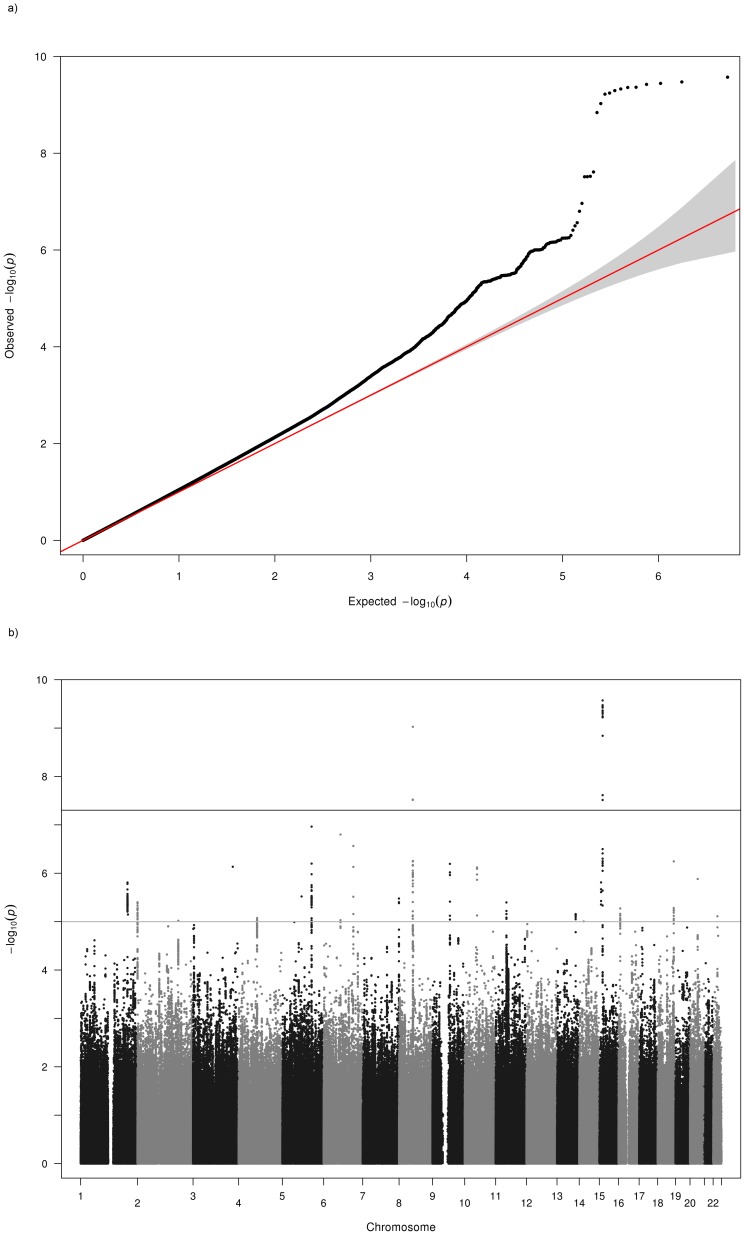
Q-Q and Manhattan Plots for the hyperopia analysis excluding the ERF cohort a) Q-Q plot for association between all SNPs analyzed and hyperopia in the meta-analysis excluding the ERF cohort. Each dot represents an observed statistic (defined as -log10 P) versus the corresponding expected statistic. The red line corresponds to the null distribution. b) Manhattan plot for association between all SNPs analyzed and hyperopia in the meta-analysis excluding the ERF cohort. Each dot represents an observed statistic (defined as -log10 P). The darker gray line corresponds to the genome-wide significance threshold and the lighter gray line represents the suggestive threshold.

Due to the high genomic control values for OGP-Talana and ERF (Table 1), we examined QQ plots of only the common SNPs (MAF>0.2) to see if this made an improvement, since all the SNPs reported here have high MAFs. In OGP-Talana this improved the QQ plots (Figure S9) but it made no difference for ERF. Therefore, we dropped ERF from the analysis and re-examined the results (Figure 4). For all loci this made minimal difference to the p values and did not change the conclusions.

## Discussion

We conducted a meta-analysis of 9 myopia and hyperopia genome-wide association studies. We detected the known loci on chromosomes 8q12 and 15q14. The locus on chromosome 8q12 has been reported associated with mean spherical equivalent in an analysis which included many of the cohorts in this study [19], and myopia age at onset in an independent study [18]. The locus on chromosome 15q14 was discovered in some of the cohorts included in this analysis [48] and has been well replicated in studies of both MSE [21] and myopia age at onset [18]. These findings were therefore expected. However, the signal for 15q14 is only genome-wide significant in the hyperopia analysis here. In addition, although the 8q12 locus was genome-wide significant in the myopia analysis, it was more significant in the hyperopia analysis. Nonetheless, the direction of effect of these SNPs is exactly opposite in the myopia and hyperopia analyses – suggesting that the causal mechanisms being tagged by these SNPs are operating across the spectrum of refractive error.

We also examined the results of our discovery meta-analyses of myopia (which were adjusted for age at examination and years of education) to attempt targeted “replication” of 35 GWAS-identified loci that have previously been reported by Kiefer *et al.* to be associated with age at onset of myopia [18]. Since age at onset was not available in all our study samples, it was not possible to perform an exact replication of the Kiefer *et al.* [18] trait on which they performed survival analysis of myopia age at onset. Our analyses, where we included age at exam and years of education, is the closest phenotype we had available. We also examined evidence for association with hyperopia in these same regions of the genome, since myopia and hyperopia represent opposite ends of the distribution of refractive error. It is reasonable that loci that affect the variability of MSE as a whole may therefore affect risk of both myopia and hyperopia.

Our analysis provides evidence for replication of a number of loci identified by Kiefer *et al.* [18]. Those which were replicated using the myopia trait (Table 2) represent the closest phenotype available from all of our samples to the one used in their analysis. In particular, this study presents the first report of replication of 11 regions associated with myopia. Of note, nine of these regions also showed genome-wide significant evidence of association to MSE by Verhoeven *et al.* [49]: chromosome 2 near *PRSS56* (MIM: 609995), chromosome 4 near *BMP3* (MIM:112263), chromosome 6 near LAMA2 (MIM:156225), chromosome 8 near *ZMAT4* (40734662 bp), chromosome 8 near *TOX* (MIM:606863, 60178580 bp), chromosome 10 near *BICC1* (MIM: 612717), chromosome 13 near *ZIC2*(MIM:603073)/*ZIC5*, chromosome 15 near *GJD2* (MIM:607058) and chromosome 16 near *RBFOX1*(MIM:605104). The candidate genes in these 9 regions have been discussed by both Kiefer *et al.* [18] and Verhoeven *et al.* [19]. The two remaining Kiefer et al. loci that were not reported as significantly associated with MSE in Verhoeven *et al.* [19] were on 3p26.1 and 6q13. The SNP reported by Kiefer *et al.* [18] in the 3p26.1 region did not meet our replication threshold but another SNP, only 256bp away and in strong linkage disequilibrium with this SNP, did meet our threshold. Kiefer *et al.* [18] proposed the nearby gene *SETMAR* (MIM:609834), a histone methylation and DNA repair gene as a candidate to explain their observed association with myopia. However, both the SNP detected in our study and the SNP reported by Kiefer *et al.* [18] are intronic to one transcript of *SUMF1* (MIM:607939), which codes for an enzyme that catalyzes the hydrolysis of sulfate esters. Mutations in this gene are known to cause the lysosomal storage disorder multiple sulfatase deficiency. This multisystem syndrome has been reported to have ocular phenotypes, in the form of retinal degeneration and nystagmus [50]. However, this signal on 3p26.1 was no longer a significant replication when the ERF study results were removed from the analysis. While the Q-Q plot of the ERF study results shows some deviation from expected, it does not appear to exhibit overall inflation of the false positive rate for this sample. Thus the replication of this 3p26 locus using all 9 studies may be valid but additional evidence from a larger study will be useful in determining the importance of this locus to risk of myopia. In the 6q13 region, our study replicated the exact same SNP that was reported to have the strongest association with myopia age at onset in the Kiefer *et al.* [18] study and this result did not change with the removal of the ERF study results from our meta-analysis. This associated SNP is in an intron of the *KCNQ5* gene (potassium voltage-gated channel, KQT-like subfamily, member 5, MIM:607357), which is a member of the *KCNQ* potassium channel gene family. *KCNQ5* has been shown to be differentially expressed in subregions of the brain and in skeletal muscle [51]. Voltage-dependent potassium channels are important regulators of the resting membrane potential and affect the excitability of electrically active cells (MIM: 607357). *KCNQ5* is also expressed in the retinal pigment epithelium (RPE) and neural retina. These potassium channels are believed to affect ion flow across the RPE [52] and the function of cone and rod photoreceptors [52], [53].

Other regions that were found to be significantly associated with myopia by Kiefer *et al.* [18] showed some evidence of association with hyperopia but not with myopia in our data. The significance levels of these associations reached our “replication” threshold. This intriguing result suggests that these loci may not be myopia specific. However, much larger sample sizes will be required to further investigate this issue.

One of the Kiefer *et al.* [18] loci that did not replicate in the analysis of myopia and was not previously reported as significantly associated with MSE was a locus on 2q31.2. This locus showed evidence of association with hyperopia in our data that reached our “replication” threshold. Kiefer *et al.* suggested that this association might be due to variants in the phosphodiesterase 11A gene (*PDE11A,* MIM:604961), which as a known cell signaling molecule is a good candidate gene for development of refractive errors, given the importance of neural signaling in the control of eye growth. However, the signal in our hyperopia analysis stretches across 3 genes: *PDE11A*; tetratricopeptide repeat domain 30A (*TTC30A*) protein; and alkylglycerone phosphate synthase (*AGPS*, MIM:603051). Mutations in AGPS are associated with rhizomelic chondrodysplasia punctata, type 3, a multisystem developmental disorder in which patients frequently develop cataracts [54].

For the locus on chromosome 4 that showed some evidence of association with hyperopia in our data, Kiefer *et al.* [18] suggested that *ANTXR2* (MIM:106490), a gene involved in extracellular matrix adhesion was the best candidate, but other good candidates exist in this region such as BMP2 inducible kinase (*BMP2K*) and annexin A3 (*ANXA3*, MIM:106490) a gene involved in regulation of cell growth and signal transduction pathways. Two other bone morphogenic proteins whose genes are located elsewhere in the genome have been identified as candidate genes by Kiefer *et al.* [18] and Verhoeven *et al.* [19] and have also been observed in animal models of myopia [55], [56]. The role of this group of genes in growth regulation is well known [57].

Given that hyperopia and myopia are the extreme ends of the refractive error distribution, it is tempting to assume that the same risk factors must affect the risk of developing both traits equally. However, it is not yet clear whether those environmental and genetic factors which increase the risk of developing myopia necessarily affect the risk of hyperopia. The results presented here provide some tantalizing evidence that some genetic factors may be important in both traits whereas others may be more important in driving myopization than hyperopization or *vice versa*. It has now been shown that 9 regions (2q37, 4q21, 6q22, 8p11, 8q12, 10q21, 13q32, 15q14, 16p13) show association to age at onset of myopia [18], myopia adjusted for age at exam, sex and years of education (results presented here) and mean spherical equivalent [19]. However, we observed replication-level association with myopia for an additional 2 loci (6q13 and 8p11) which were not genome-wide significant for mean spherical equivalent [19] but were genome-wide significant for myopia age at onset [18]. An additional four regions that were genome-wide significant in the Kiefer *et al.* analysis of age at onset of myopia [18] have only been “replicated” in our hyperopia analyses. These results indicate that the genetic underpinnings of refractive errors are quite complex and that analyses of both the qualitative and quantitative phenotypes may add to our understanding of refractive error causation. The study participants whose data were analyzed here were not selected for extreme or “high” myopia (typically defined as SE <-6D) and there were very few individuals with high myopia in any of these datasets. Future studies to examine whether any of the loci that show association to myopia, hyperopia and mean spherical equivalent in the population-based studies also show evidence of association to high myopia would be interesting and should be pursued.

Some of the other loci that showed significant association with myopia in the Kiefer *et al.* [18] study did not replicate in our current study. Dichotomizing the trait from spherical equivalent to myopia or hyperopia in each population did reduce sample size for each population compared to the number of individuals with measurements of spherical equivalent. This consequent reduction in power was the reason we added additional populations to our discovery meta-analysis compared to our refractive error meta-analysis [24], to offset the lower sample size. This current study is still, however, smaller than the Kiefer et al. [18] study we were attempting to replicate and so some of the other loci may yet replicate in a larger study.

In summary, we have provided evidence in favor of replication of 11 loci involved in causation of myopia. Twelve loci that have been shown to be associated with myopia age at onset [18] showed “replication-level” association with hyperopia here (7 of these loci also showed replication-level association with the myopia trait; 5 loci only showed this level of association with hyperopia). Further research is required to determine whether any of the candidate genes identified near these associated SNPs are truly causing the development of refractive errors, or whether the actual causal variant is located in another nearby gene or other functional locus in high LD with the SNPs associated with the trait. Evidence for expression of many of these genes have indicated that they are active in the eye [19] and investigation of the ENCODE data suggests many loci have regulatory functions, which is consistent with the current hypothesis of regulation of eye growth through a visually-evoked signaling cascade. However, more research using in vitro and in vivo models is necessary to elucidate the underlying mechanisms of normal emmetropization and how it can be disrupted to produce refractive errors.

## Supporting Information

Figure S1 **QQ and Genome-wide Manhattan plot of genotyped and imputed SNPs for association with myopia (A,C) and hyperopia (B,D) in AREDS.**
(TIF)

Figure S2 **QQ and Genome-wide Manhattan plot of genotyped and imputed SNPs for association with myopia (A,C) and hyperopia (B,D) for KORA.**
(TIF)

Figure S3 **QQ and Genome-wide Manhattan plot of genotyped and imputed SNPs for association with myopia (A,C) and hyperopia (B,D) for Framingham Eye Study.**
(TIF)

Figure S4 **QQ and Genome-wide Manhattan plot of genotyped and imputed SNPs associated with myopia (A,C) and hyperopia (B,D) in MESA.**
(TIF)

Figure S5 **QQ and Genome-wide Manhattan plot of genotyped and imputed SNPs for association with myopia (A,C) and hyperopia (B,D) in OGP-Talana.**
(TIF)

Figure S6 **QQ and Genome-wide Manhattan plot of genotyped and imputed SNPs for association with myopia (A,C) and hyperopia (B,D) in ERF.**
(TIF)

Figure S7 **QQ and Genome-wide Manhattan plot of genotyped and imputed SNPs for association with myopia (A,C) and hyperopia (B,D) in RS-I.**
(TIF)

Figure S8 **QQ and Genome-wide Manhattan plot of genotyped and imputed SNPs for association with myopia (A,C) and hyperopia (B,D) in RS-II.**
(TIF)

Figure S9 **QQ and Genome-wide Manhattan plot of genotyped and imputed SNPs for association with myopia (A,C) and hyperopia (B,D) in RS-III.**
(TIF)

Figure S10 **Flowchart showing the analysis workflows of the entire study.**
(TIF)

Table S1 **Chromosomal regions selected to represent loci reported to exhibit genome-wide significant or suggestive association with myopia age at onset by Kiefer** ***et al.*** **(2013).**
(XLSX)

Table S2 **Association results in our discovery meta-analysis for the complete set of SNPs selected to represent loci reported to exhibit genome-wide significant or suggestive association with myopia age at onset by Kiefer et al. (2013).**
(XLSX)

Table S3 **Baseline Characteristics of Samples Used in the Replication of Our Discovery Meta-analysis Results.**
(XLSX)

Table S4 **Most significant associations with myopia in the discovery GWAS meta-analysis.**
(XLSX)

Table S5 **Most significant associations with hyperopia in the discovery GWAS meta-analysis.**
(XLSX)

Table S6 **Original Myopia Discovery Meta-analysis p-value and Replication p-value from Meta-analysis of Regional Results in 8 Replication Samples.**
(XLSX)

Table S7 **Original Hyperopia Discovery Meta-analysis p-value and Replication p-value from Meta-analysis of Regional Results in 8 Replication Samples.**
(XLSX)

Table S8 **Comparison of minor allele frequencies (MAF) for each of the Kiefer et al. SNPs for the discovery populations.**
(XLSX)

Checklist S1 **PRISMA Checklist.**
(DOCX)

Materials S1 **Replication Study Participants, Genotyping, Quality Control and Imputation.**
(DOCX)

Materials S2 **Association Analysis of Discovery Samples.**
(DOCX)

Materials S3 **Supplementary References.**
(DOCX)
