# Supplementary figures and images for: Genome-Wide Meta-Analysis of Myopia and Hyperopia Provides Evidence for Replication of 11 Loci
Source: PLoS One. 2014 Sep 18;9(9):e107110. doi: 10.1371/journal.pone.0107110 (PMC4169415; doi:10.1371/journal.pone.0107110)

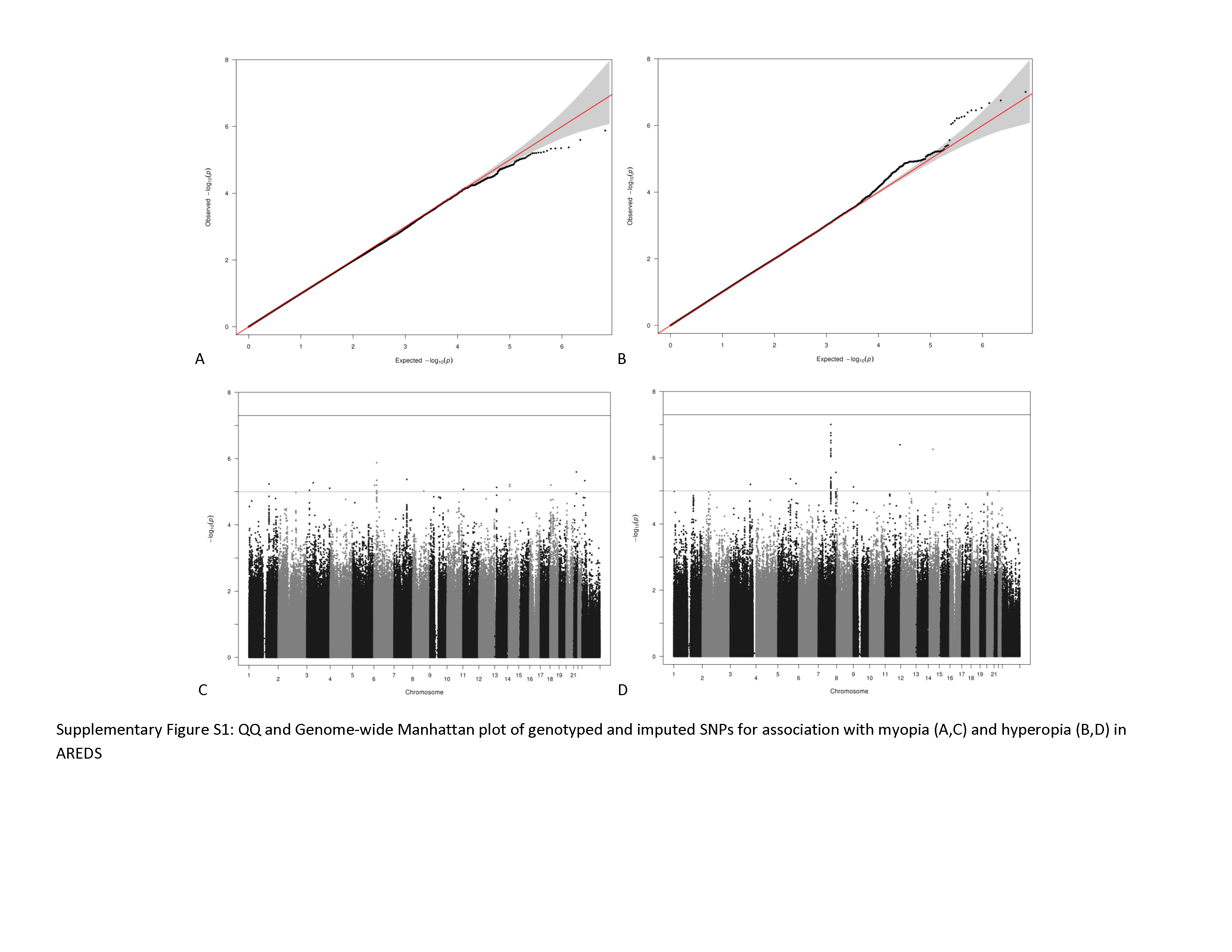

Supplement: Figure S1 — QQ and Genome-wide Manhattan plot of genotyped and imputed SNPs for association with myopia (A,C) and hyperopia (B,D) in AREDS. (TIF) [file pone.0107110.s001.tif]

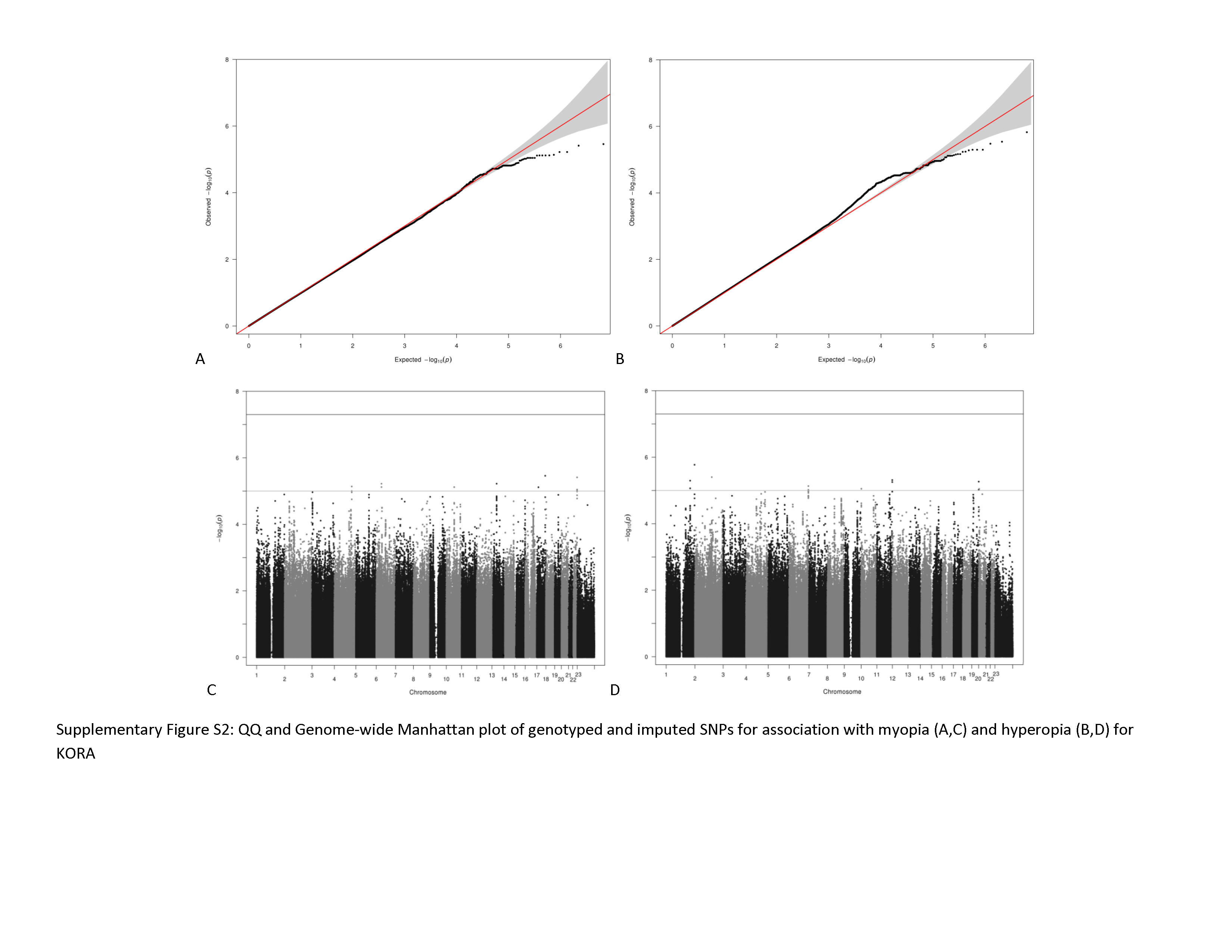

Supplement: Figure S2 — QQ and Genome-wide Manhattan plot of genotyped and imputed SNPs for association with myopia (A,C) and hyperopia (B,D) for KORA. (TIF) [file pone.0107110.s002.tif]

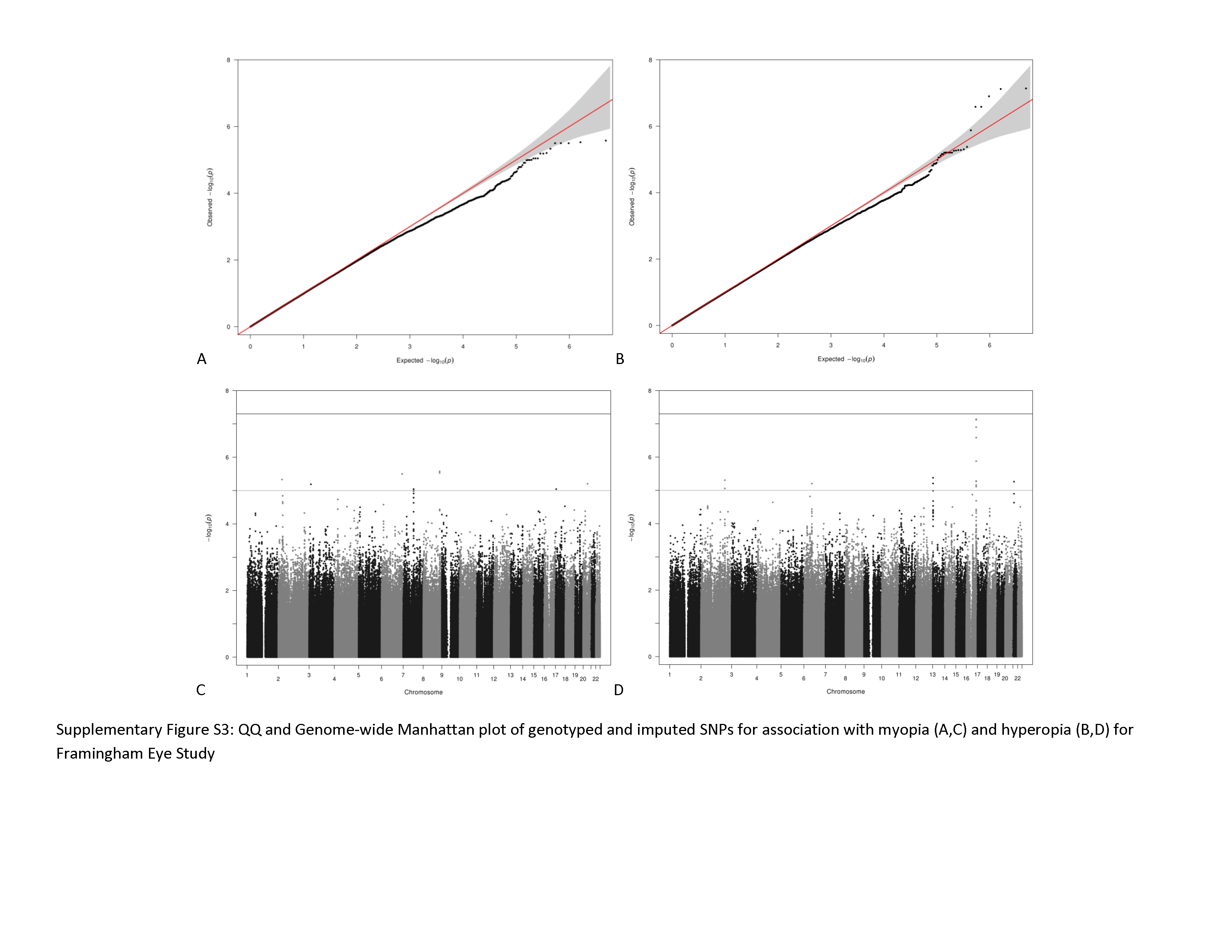

Supplement: Figure S3 — QQ and Genome-wide Manhattan plot of genotyped and imputed SNPs for association with myopia (A,C) and hyperopia (B,D) for Framingham Eye Study. (TIF) [file pone.0107110.s003.tif]

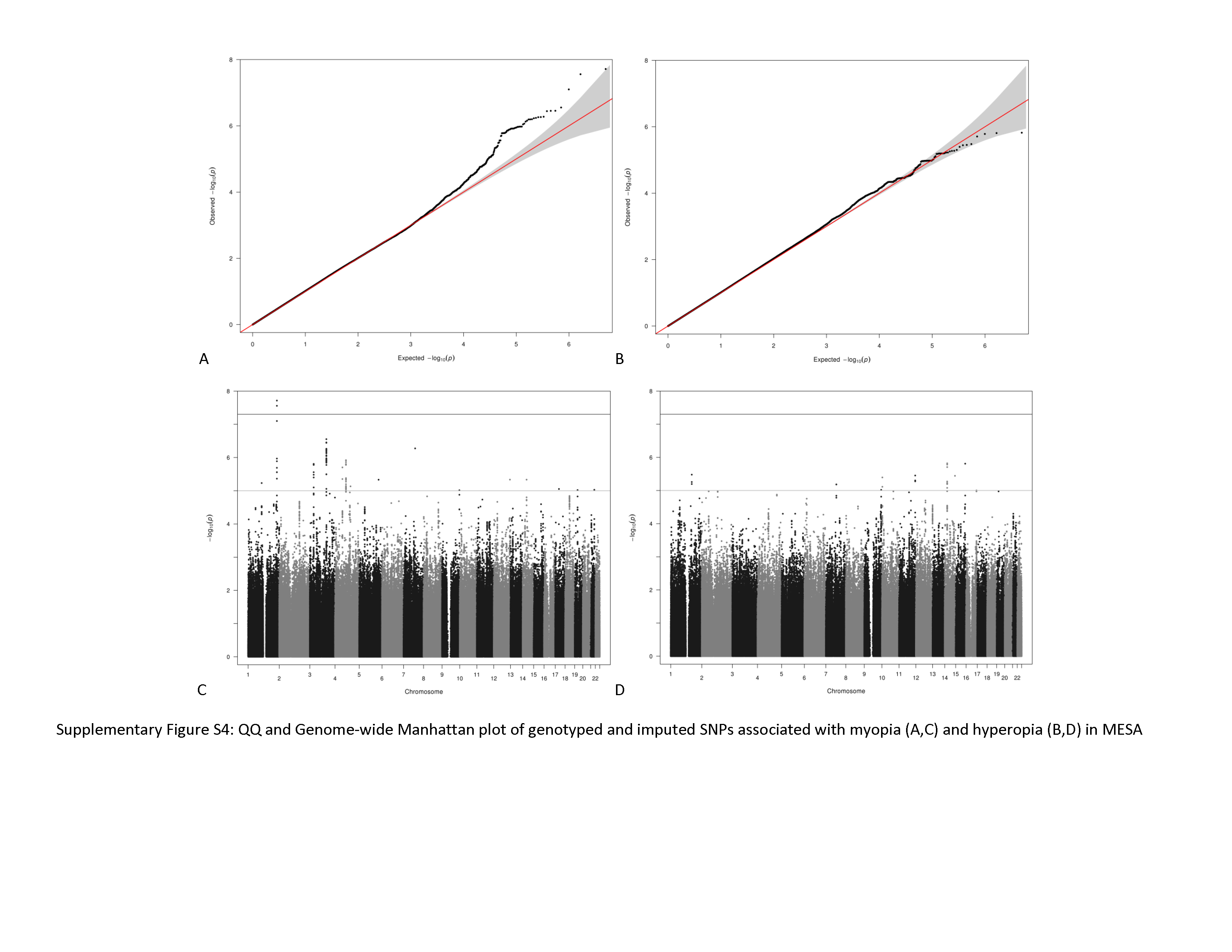

Supplement: Figure S4 — QQ and Genome-wide Manhattan plot of genotyped and imputed SNPs associated with myopia (A,C) and hyperopia (B,D) in MESA. (TIF) [file pone.0107110.s004.tif]

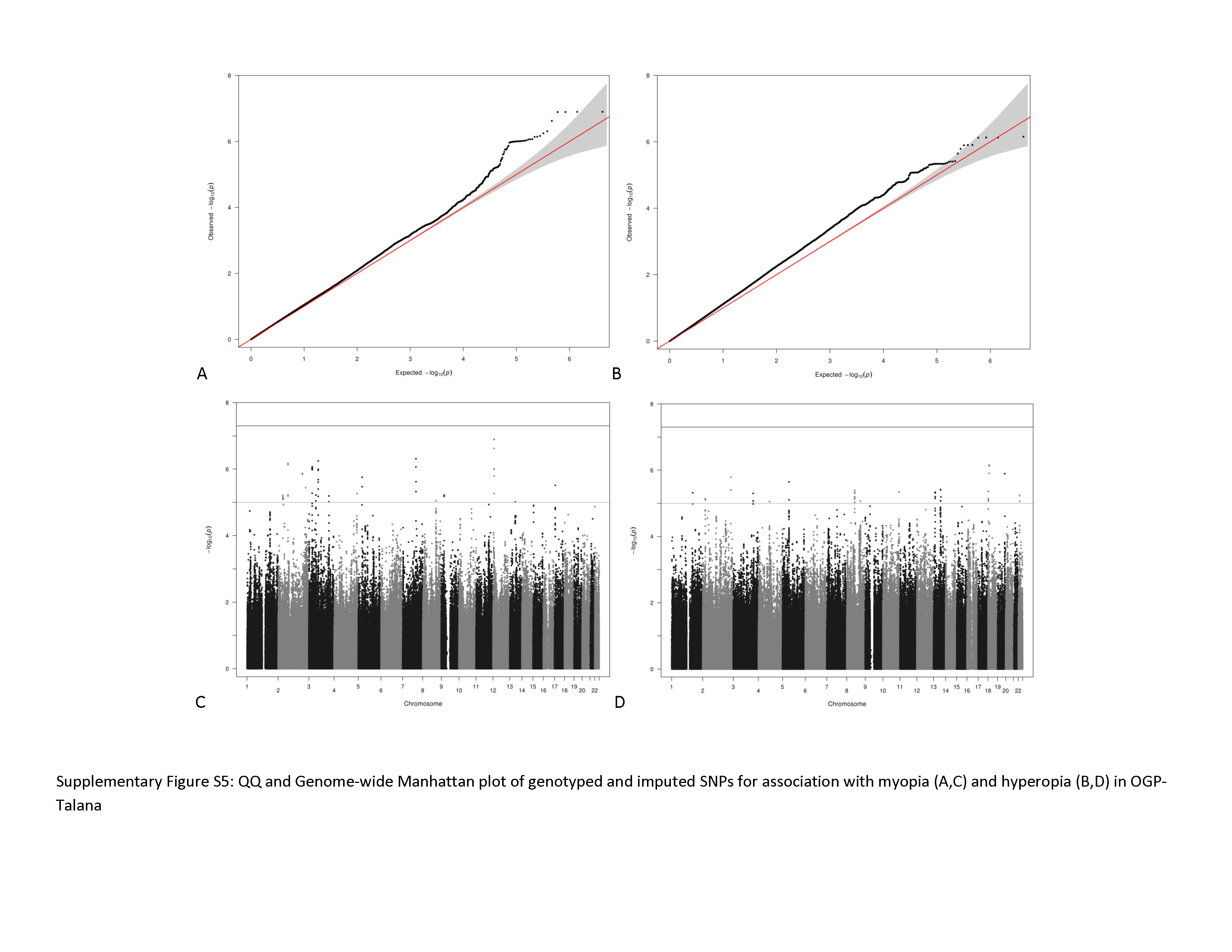

Supplement: Figure S5 — QQ and Genome-wide Manhattan plot of genotyped and imputed SNPs for association with myopia (A,C) and hyperopia (B,D) in OGP-Talana. (TIF) [file pone.0107110.s005.tif]

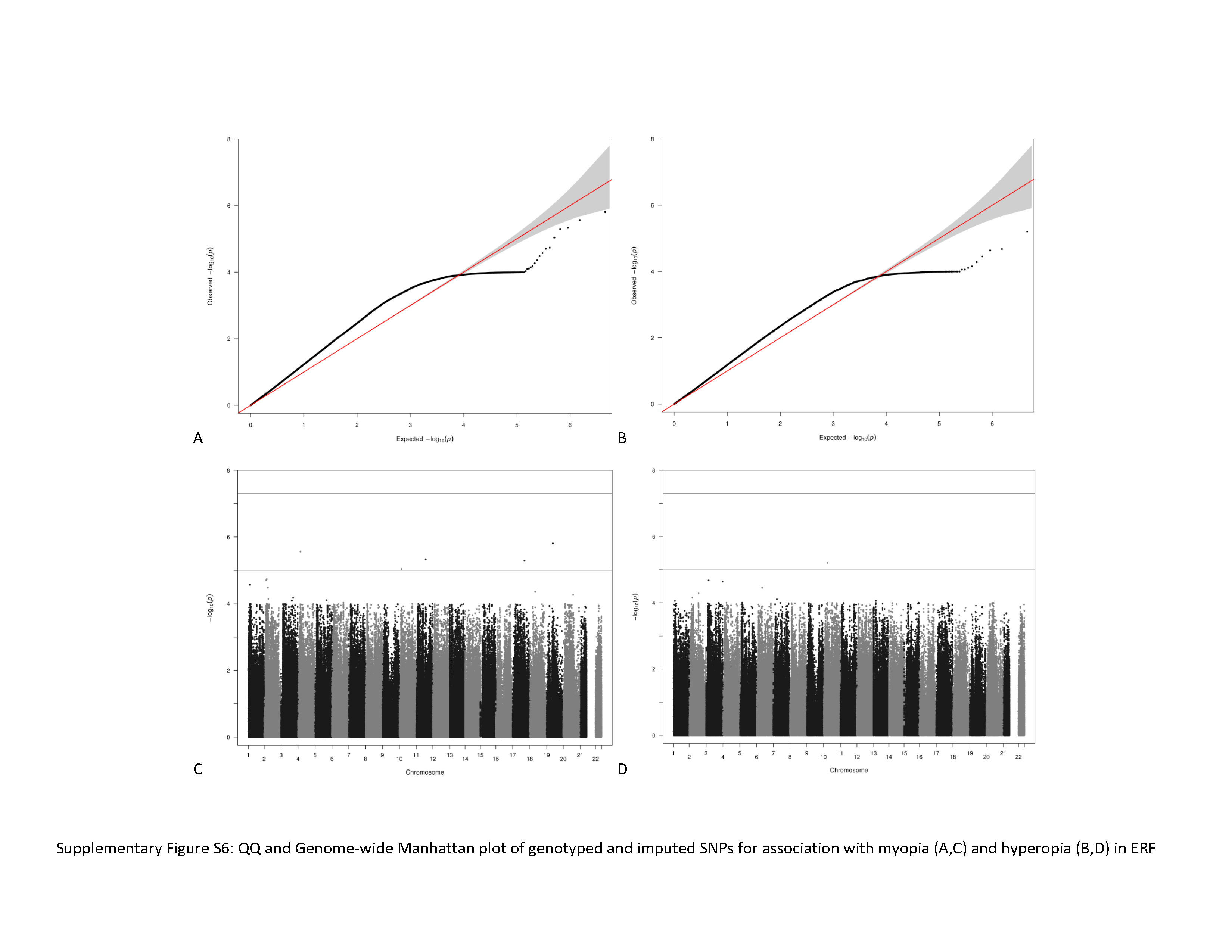

Supplement: Figure S6 — QQ and Genome-wide Manhattan plot of genotyped and imputed SNPs for association with myopia (A,C) and hyperopia (B,D) in ERF. (TIF) [file pone.0107110.s006.tif]

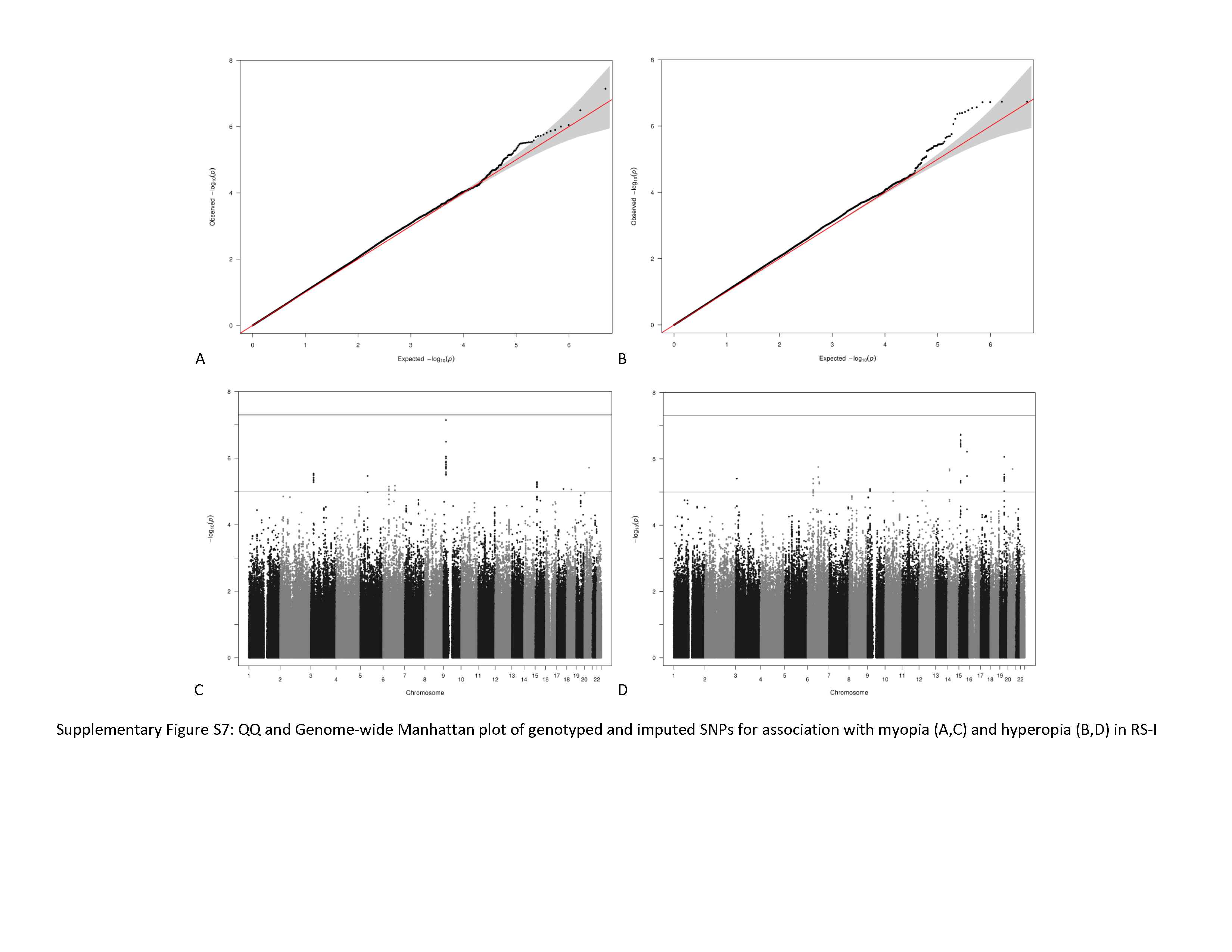

Supplement: Figure S7 — QQ and Genome-wide Manhattan plot of genotyped and imputed SNPs for association with myopia (A,C) and hyperopia (B,D) in RS-I. (TIF) [file pone.0107110.s007.tif]

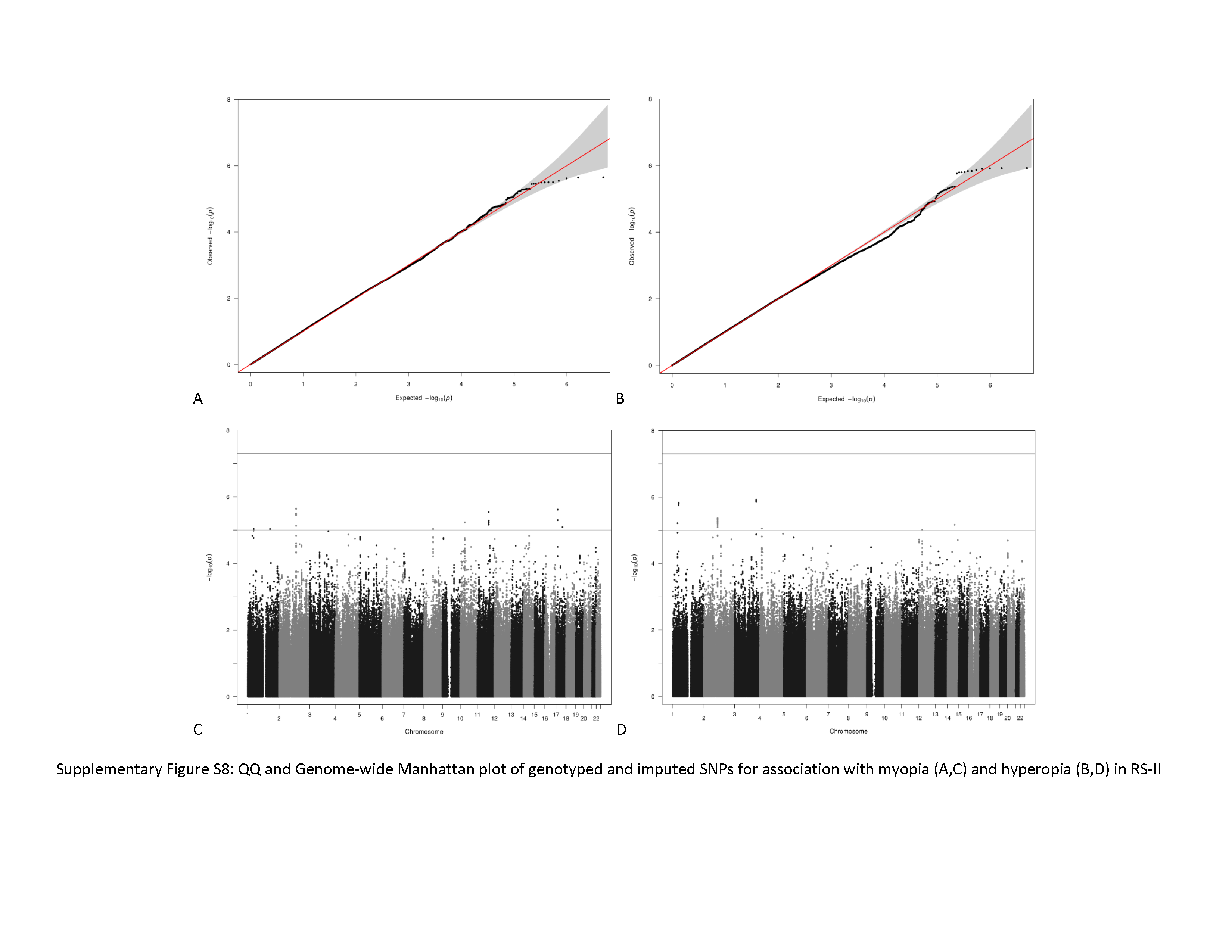

Supplement: Figure S8 — QQ and Genome-wide Manhattan plot of genotyped and imputed SNPs for association with myopia (A,C) and hyperopia (B,D) in RS-II. (TIF) [file pone.0107110.s008.tif]

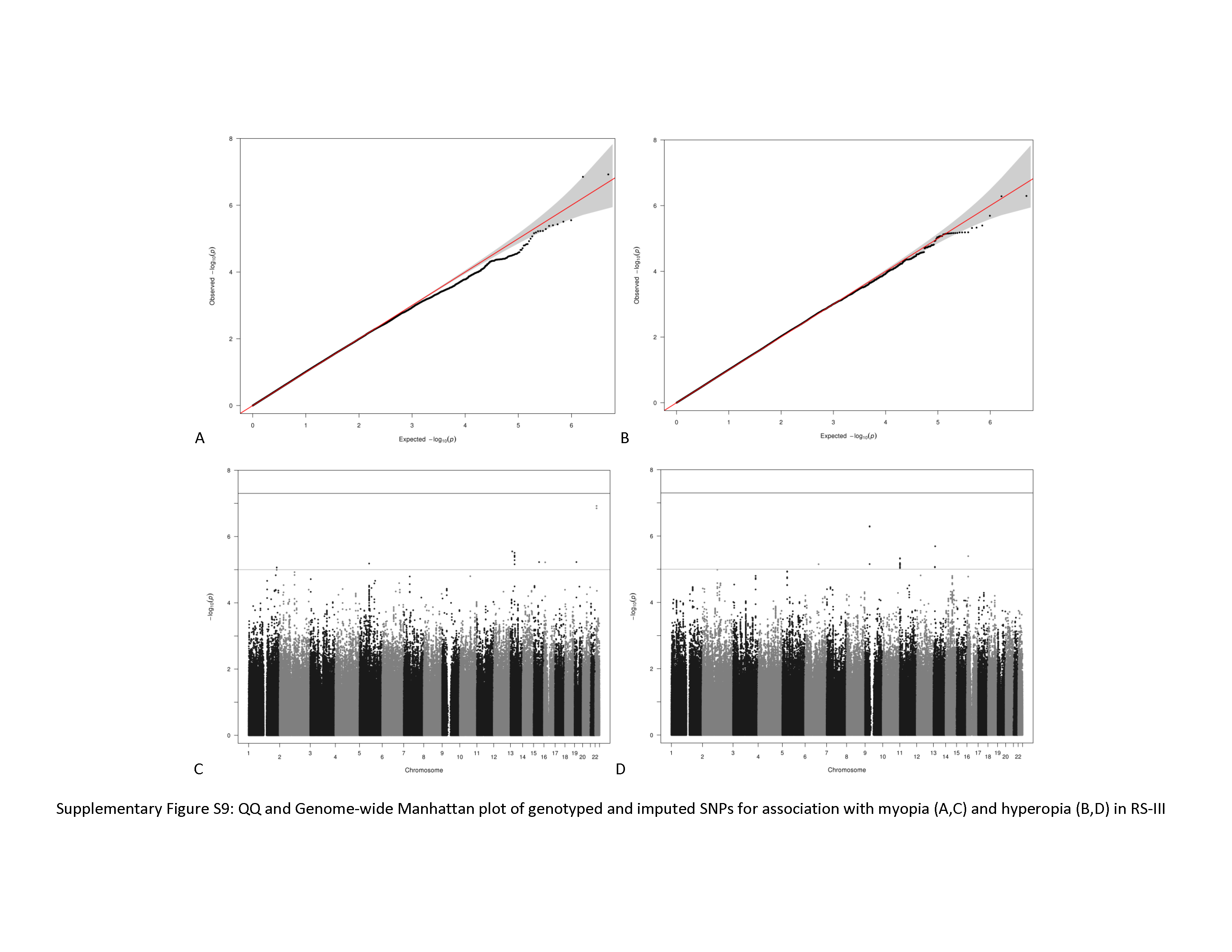

Supplement: Figure S9 — QQ and Genome-wide Manhattan plot of genotyped and imputed SNPs for association with myopia (A,C) and hyperopia (B,D) in RS-III. (TIF) [file pone.0107110.s009.tif]

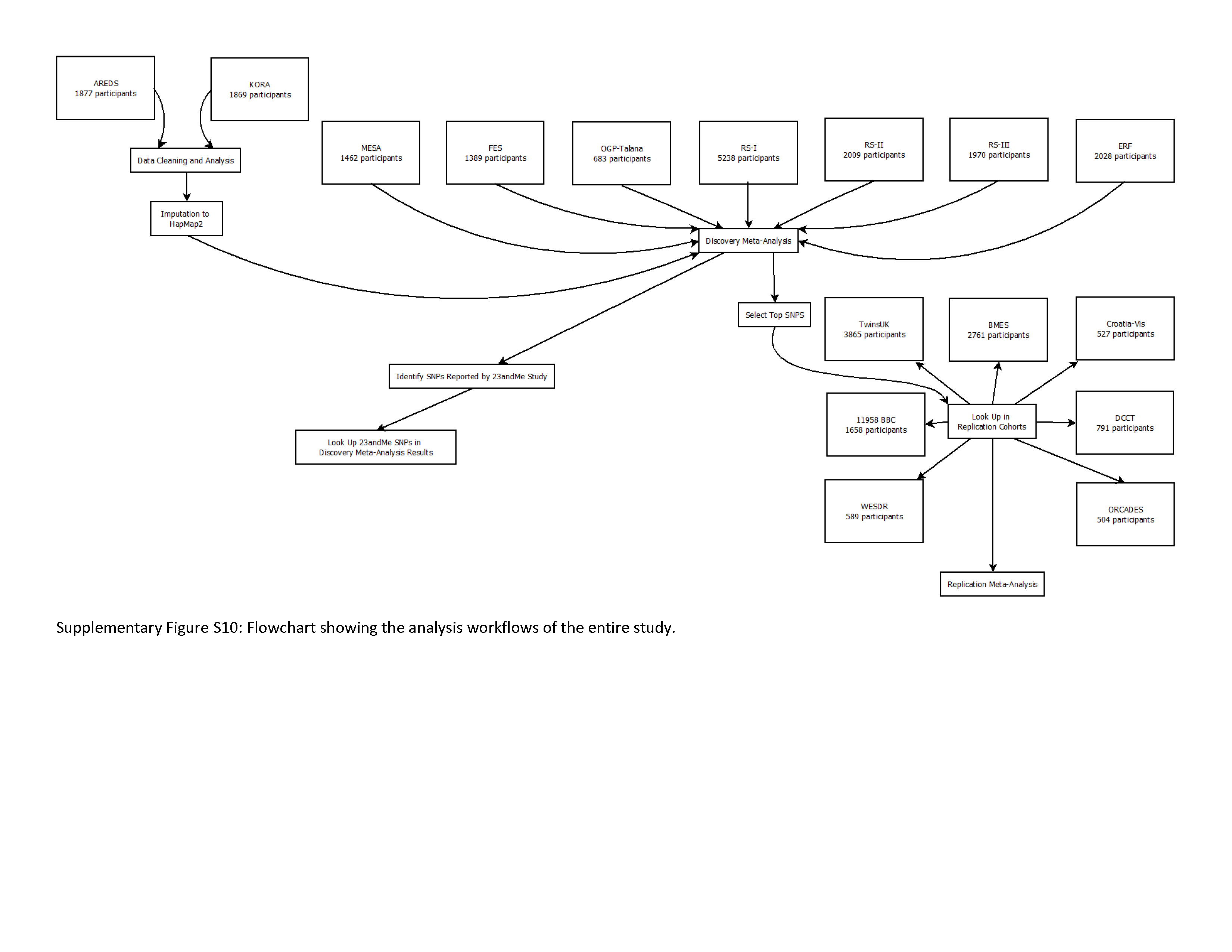

Supplement: Figure S10 — Flowchart showing the analysis workflows of the entire study. (TIF) [file pone.0107110.s010.tif]
