# Supplementary material for: Genome-Wide Meta-Analysis of Myopia and Hyperopia Provides Evidence for Replication of 11 Loci
Source: PLoS One. 2014 Sep 18;9(9):e107110. doi: 10.1371/journal.pone.0107110 (PMC4169415; doi:10.1371/journal.pone.0107110)
Supplement: Materials S1 — Replication Study Participants, Genotyping, Quality Control and Imputation. (DOCX) [file pone.0107110.s020.docx]

# SUPPLEMENTAL MATERIALS S1: REPLICATION STUDY PARTICIPANTS, GENOTYPING, QUALITY CONTROL AND IMPUTATION

***1958 British Birth Cohort***

The 1958 British Birth Cohort ([1](#_ENREF_1)) is a prospective population‐based cohort study that initially included 17,000 newborn children whose birth was within the first week of March 1958. All participants gave informed written consent to participate in genetic association studies, and the study was approved by the South East Multi Centre Research Ethics Committee (MREC) and the Oversight Committee for the biomedical examination of the British 1958 British birth cohort. Biomedical examination protocols were approved by the South East MREC. Assessment of refraction was undertaken in a random subsample of cohort members through non‐cycloplegic autorefraction (Nikon Retinomax 2) of both eyes of each subject. Illumina’s Human1M‐Duo chip was used for genotyping. Imputation was calculated with reference to HapMap release 22 CEU population data using IMPUTE version 2. Individuals were checked for genotyping success rate (all exceeded 99%), excess or low heterozygosity (all participating subjects were checked and found within the pre‐defined interval of 0.2‐0.4). SNPs were included in the analysis if they had a genotype success rate of at least 0.95, were within Hardy‐Weinberg equilibrium (p>10^‐04^) and had a minor allele frequency of 0.04 or above. Counts of cases and controls and genomic control values are given in Supplemental Table S3. A logistic regression model was implemented in PLINK using sex as covariate.

***Blue Mountains Eye Study (BMES)***

The Blue Mountains Eye Study (BMES) is a population-based cohort of a predominantly white population in west of Sydney, Australia. Over 98% of BMES participants were European ancestry. All BMES examinations were approved by the Human Ethics Committees of the Western Sydney Area Health Service and University of Sydney. Signed informed consent was obtained from participants at each examination.
Participants of the BMES cross section II who had DNA available in early 2009

(n=2983) were genotyped using the Illumina Human 670-Quadv1 custom genotyping array at the Wellcome Trust Sanger Institute, Cambridge as part of WTCCC2, and 2761 had genotyping data available. Following exclusion through GWAS and DNA quality control and removal of individuals who had undergone cataract surgery, had severe visual impairment or had any known ocular pathologies such as macular degeneration and nuclear cataracts resulted in genotyping data being available for 2,194 individuals.
Imputation was performed from the Hap Map using IMPUTE2.034. Imputed SNPs were excluded from the analysis when failing one or more of the following QC filters: 1) prop info ≥ 0.5 (a software-specific statistic from IMPUTE); 2) Hardy-Weinberg *P*-value < 1×10^-6^. Counts of cases and controls and genomic control values are given in Supplemental Table S3. Logistic regression was performed using PLINK including age, sex and 2 principal components in the model.

***CROATIA-Vis Island Study***

The CROATIA-Vis island study is a population-based, cross-sectional study in Croatia including adult participants, aged 18–93 years (mean = 56), a subset of which (N=640) underwent a complete eye examination in summer 2007 and provided their ophthalmologic history ([2](#_ENREF_2)). The study received approval from relevant ethics committees in Scotland and Croatia and followed the tenets of the Declaration of Helsinki. Non-cycloplegic autorefraction were measured on each eye using a NIDEK Ark30 hand-held autorefractometer. Measures on eyes with a history of trauma, intra-ocular surgery or LASIK operations were removed and the analysis was done on the right eye measures, unless the left eye had more complete measurements (e.g. due to trauma or cataract surgery on the right eye). Extreme values (lying more than 3 interquartile range from the upper or lower quartile) were removed. Genotypes were generated using a dense Illumina SNP array, 370CNV-Quad, following the manufacturer’s standard recommendations. Genotypes were determined using the Illumina BeadStudio software. Samples with a call rate below 97 % , potentially mixed samples with excess autosomal heterozygosity or gender discrepancy (based on the sex chromosomes genotypes), and ethnic outliers (based on principal components analysis of genotypic data), were excluded from the analysis using the quality control algorithm implemented in the R package GenABEL. Imputation of allele dosage for over 2 millions SNPs on the 22 autosomal chromosomes with reference to HapMap CEU build 36 release 22 was performed using the software MACH v1.0.15 after exclusion of SNP with MAF < 0.01, call rate < 98% and HWE deviation p< 10^-6^. Counts of cases and controls and genomic control values are given in Supplemental Table S3. Genome-wide association analysis was performed using the ProbABEL package using an additive SNP allelic effect model and correcting for individual relatedness using the polygenic and mmscore functions implemented in the GenABEL package. To accomplish this, two ProbABEL analyses are run, one using the palogistic function which gives estimates of OR and the second using the palinear function with the outcome as a quantitative trait and correcting for relatedness which yields corrected p-values of association upon which the standard errors of OR estimates are calculated.

***CROATIA-Korcula Study***

The CROATIA-Korcula study is a population-based, cross-sectional study in Croatia that includes a total of 969 adult examinees, aged 18-98 (mean=56.3) from the Dalmatian island of *Kor*č*ula*, and most (N=930) underwent a complete eye examination ([2](#_ENREF_2))}.The study received approval from relevant ethics committees in Scotland and Croatia and followed the tenets of the Declaration of Helsinki. Non-cycloplegic autorefraction were measured on each eye using a NIDEK Ark30 hand-held autorefractometer. Measures on eyes with a history of trauma, intra-ocular surgery or LASIK operations were removed and the analysis was done on the right eye measures, unless the left eye had more complete measurements (e.g. due to trauma or cataract surgery on the right eye). Extreme values (lying more than 3 interquartile range from the upper or lower quartile) were removed.. Genotypes were generated using a dense Illumina SNP array, 370CNV-Quad, following the manufacturer’s standard recommendations. Genotypes were determined using the Illumina BeadStudio software. Samples with a call rate below 97 % , potentially mixed samples with excess autosomal heterozygosity or gender discrepancy (based on the sex chromosomes genotypes), and ethnic outliers (based on principal components analysis of genotypic data), were excluded from the analysis using the quality control algorithm implemented in the R package GenABEL. Imputation of allele dosage for over 2 millions SNPs on the 22 autosomal chromosomes with reference to HapMap CEU build 36 release 22 was performed using the software MACH v1.0.15 after exclusion of SNP with MAF < 0.01, call rate < 98% and HWE deviation p< 10^-6^. Counts of cases and controls and genomic control values are given in Supplemental Table S3. Genome-wide association analysis was performed using the ProbABEL package using an additive SNP allelic effect model and correcting for individual relatedness using the polygenic and mmscore functions implemented in the GenABEL package. To accomplish this, two ProbABEL analyses are run, one using the palogistic function which gives estimates of OR and the second using the palinear function with the outcome as a quantitative trait and correcting for relatedness which yields corrected p-values of association upon which the standard errors of OR estimates are calculated.

***Diabetes Control and Complications Trial***

The Diabetes Control and Complications Trial (DCCT, 1989-1993) was a multi-center study of patients with type 1 diabetes from the United States and Canada. All the participants went through complete ophthalmologic examinations at the study baseline and every year thereafter (average 6 visit per individual). The ophthalmologic visits included slit lamp examination, direct ophthalmoscopy, measurements of intraocular pressure and visual acuity. Stereoscopic fundus photographs were also taken every 6 months during the study. As part of the year ophthalmologic examination, subjective refraction (sphere, cylinder and axis) was measured using a chart at 10 to 20 feet. Education level was recorded at baseline visit (F002) and updated yearly. Most of the participants were followed in EDIC (Epidemiology of Diabetes Interventions and Complications) and have gone through an average of 4 more ophthalmologic exams during a 15 year follow-up period. Exclusion criteria included cataracts and past history of cataract or refractive surgery. Myopia status was defined based on subjective refraction at baseline visit ophthalmologic exam. Counts of cases and controls and genomic control values are given in Supplemental Table S3.

Participants were genotyped using the Illumina Human 1M beadchip assay. Individuals were excluded from subsequent analysis based on the following criteria: discrepancies between reported sex and genotype data (n=3); genotype call rate less than 98% (no data removed, minimum call rate threshold = 0.988); disagreements between genotypes of SNPs with an earlier study (n=58). To detect cryptic relatedness and/or sample mix-ups, IBS estimates between all pairs of individuals were performed and two probands were removed. No individuals were removed due to sample contamination assessed by calculation of the mean heterozygosity across the genome for each individual. The analysis was performed on the individuals who self-identified as Caucasian. Individuals who were determined to be admixed between Caucasian and other ethnic groups through population genetic approaches were excluded. SNPs were excluded based on the following criteria: Illumina Gencall score for the quality of genotyping (<0.15), autosomal SNPs showing significant association with gender (p<10^-8^), deviation from Hardy-Weinberg equilibrium (p<10-8). Genotype of 2.5M SNPs was imputed using phase 2 of HapMap as reference panel (CEU population, release 22, build 36) by Markov Chain Haplotyping method (MACH version 1.0.16) software. Association analysis was performed using logistic regression in PLINK v1.07 adjusting for age, gender, level of education and the first three principal components.

***Orkney Complex Disease Study (ORCADES)***

The Orkney Complex Disease Study (ORCADES) is a population-based, cross-sectional study in the Scottish archipelago of Orkney, including 1,285 individuals with eye measurements. The study received approval from relevant ethics committees in Scotland and followed the tenets of the Declaration of Helsinki. Autorefractive measurements were obtained using a Kowa KW 2000 autorefractometer. Measures on eyes with a history of trauma, intra-ocular surgery or LASIK operations were removed and the analysis was done on the right eye measures, unless the left eye had more complete measurements (e.g. due to trauma or cataract surgery on the right eye) ([3](#_ENREF_3)). Extreme values (lying more than 3 interquartile range from the upper or lower quartile) were removed. Genotypes were generated using a dense Illumina SNP arrays, HumanHap 300v2 and 370CNV-Quad, following the manufacturer’s standard recommendations. Genotypes were determined using the Illumina BeadStudio software. Samples with a call rate below 97 % , potentially mixed samples with excess autosomal heterozygosity or gender discrepancy (based on the sex chromosomes genotypes), and ethnic outliers (based on principal components analysis of genotypic data), were excluded from the analysis using the quality control algorithm implemented in the R package GenABEL. Imputation of allele dosage for over 2 millions SNPs on the 22 autosomal chromosomes with reference to HapMap CEU build 36 release 22 was performed using the software MACH v1.0.15 after exclusion of SNP with MAF < 0.01, call rate < 98% and HWE deviation p< 10^-6^. Counts of cases and controls and genomic control values are given in Supplemental Table S3. To accomplish this, two ProbABEL analyses are run, one using the palogistic function which gives estimates of OR and the second using the palinear function with the outcome as a quantitative trait and correcting for relatedness which yields corrected p-values of association upon which the standard errors of OR estimates are calculated.

***The TwinsUK Study***

The TwinsUK adult twin registry based at St. Thomas’ Hospital in London is a volunteer cohort of over 10,000 twins from the general population ([4](#_ENREF_4)). Twins largely volunteered unaware of the eye studies, gave fully informed consent under a protocol reviewed by the St. Thomas’ Hospital Local Research Ethics Committee and underwent non‐cyclopleged autorefraction using an ARM‐10 autorefractor (Takagi Ltd). Out of the original 4,388 subjects for whom phenotype and genotype information was available, 4,270 subjects (of whom 3865 were included in the current analysis) were included in the study; 118 subjects were excluded after failing quality control. Genotyping was carried out using three genotyping platforms from Illumina: the HumanHap 300k Duo for part of the UK Twin Cohort and the HumanHap610‐Quad array for the rest of the UK Twin Cohort. Imputation was calculated with reference to HapMap release 22 CEU population data using IMPUTE version 2. Individuals were included if their genotyping success rate exceeded 95%, did not show excess or low heterozygosity (defined by the interval interval of 0.2‐04). SNPs were included in the imputation if they had a genotype success rate of at least 0.95 if their minor allele frequency was superior to 0.005 and at least 0.99 if their MAF was 0.01‐0.05. Only SNPs that were within Hardy‐Weinberg equilibrium (p>10^‐04^) and had a minor allele frequency of 0.04 or above were regressed. Counts of cases and controls and genomic control values are given in Supplemental Table S3. A logistic regression model was implemented in PLINK using age and sex as covariates.

***Wisconsin Epidemiologic Study of Diabetic Retinopathy***WESDR is an observational cohort study of diabetes complications (1979‐2007) ([5](#_ENREF_5)). Subjective refraction, measured following standard protocols at first visit, was analyzed in the current study (n=589). Myopia status was defined based on subjective refraction at visit 1 ophthalmologic exam. Counts of cases and controls and genomic control values are given in Supplemental Table S3.

Subjects with type 1 diabetes from WESDR were genotyped using Illumina HumanOmni1‐Quad BeadChip assay. Individuals showing gender mismatch with typed X‐linked markers (n=8), cryptic relatedness (n=5), high autosomal heterozygosity (n=6), call rate <0.95 (n=30), as well as ancestries other than “white” were not included in the analysis. Population genetic approaches based on multi‐dimensional scaling implemented in PLINK v1.07 were used to identify and exclude ethnically admixed individuals. Imputation was performed in IMPUTE v2.2.2 using all populations from HapMap phase II release 22 as reference (IMPUTE2 chooses the best custom reference set for each individual 57internally). Analysis was restricted to adults (age>=18). Individuals were excluded with more than 1D difference in SE across both eyes. GWAS analysis was performed using SNPTEST software on genotype imputation data to HM2. The logistic regression adjusted for age, sex, first three PCs from PCA and number of years of education (ranged from 3 to 22 yr). SNPs with MAF<0.01 or info<0.3 (imputation quality metric) were excluded.
