# Supplementary material for: Genome-Wide Meta-Analysis of Myopia and Hyperopia Provides Evidence for Replication of 11 Loci
Source: PLoS One. 2014 Sep 18;9(9):e107110. doi: 10.1371/journal.pone.0107110 (PMC4169415; doi:10.1371/journal.pone.0107110)
Supplement: Materials S2 — Association Analysis of Discovery Samples. (DOCX) [file pone.0107110.s021.docx]

# SUPPLEMENTAL MATERIALS S2: ASSOCIATION ANALYSIS OF DISCOVERY SAMPLES

## *Age Related Eye Disease Study*

After removing all the individuals detailed above, data from 1877 AREDS participants (770 men and 1107 women) were available for analysis. The mean age was 68 years (SD=4.7; range=55-81) and the average MSE was +0.56 D (SD=2.15). Association testing was conducted on 3397980 SNPs which passed our quality control filters and had minor allele frequency (MAF) >= 0.01. The mean minor allele frequency was 0.216, the average genotyping call rate was 99.87% and none of the SNPs departed significantly from HWE. Counts of cases and controls and genomic control values are given in Table 1 of the paper.

No SNPs achieved genome-wide significance (p < 5x10^-8^) in this sample for myopia or hyperopia (Supplementary Figure 1).

## *Kooperative Gesundheitsforschung in der Region Augsburg*

After removing all the individuals detailed above, data from 1869 KORA participants (926 men and 943 women) were available for analysis. The mean age was 56 years (SD=11.8; range=35-84) and the average MSE was -0.3 D (SD=2.26). Association testing was conducted on 3390496 SNPs which passed our quality control filters and had MAF >= 0.01. The mean minor allele frequency was 0.216, the average genotyping call rate was 99.87% and none of the SNPs departed significantly from HWE. Counts of cases and controls and genomic control values are given in Table 1 of the paper.

No SNPs achieved genome-wide significance in this sample for myopia or hyperopia (Supplementary Figure 2).

## *Framingham Eye Study*

1389 individuals from the Framingham Eye Study (FES) were analyzed. The mean age was 55.6 years (SD=8.89; range=28-84) and the average MSE was 0.03 D (SD=2.41). Association testing was conducted on 2885006 SNPs which passed our quality control filters. Counts of cases and controls and genomic control values are given in Table 1 of the paper. The mean minor allele frequency was 0.23, the average genotyping call rate was 99.24% and none of the SNPs departed significantly from HWE. No SNPs achieved genome-wide significance in this sample for myopia or hyperopia (Supplementary Figure 3).

## *The Multi-Ethnic Study of Atherosclerosis*

A total of 1462 individuals (739 males and 723 females) from the Multi-Ethnic Study of Atherosclerosis (MESA) study were analyzed. The mean age was 61.9 years (SD=9.37; range=46-86) and the average MSE was -0.28 D (SD=2.62). Association testing was conducted on 2481445 SNPs which passed our quality control filters. Counts of cases and controls and genomic control values are given in Table 1 of the paper.

The mean minor allele frequency was 0.24, the average genotyping call rate was 99.84% and none of the SNPs departed significantly from HWE. Two SNPs achieved genome-wide significance in this sample for the myopia trait but none were significant for the hyperopia trait (Supplementary Figure 4).

## *Ogliastra Genetic Park - Talana study (OGP Talana)*

Six hundred eighty-three individuals from the OGP Talana study were analyzed. The mean age was 42.18 years (SD=19.14; range=5-89) and the average MSE was -0.18 D (SD=1.8. Association testing was conducted on 2093862 SNPs which passed our quality control filters. Counts of cases and controls and genomic control values are given in Table 1 of the paper.

The mean minor allele frequency was 0.5 (SD=0.28), the average genotyping call rate was 98% and none of the SNPs departed significantly from HWE. No SNPs achieved genome-wide significance in this sample (Supplementary Figure 5).

***Erasmus Rucphen Family Study***

2028 individuals from the ERF study were analyzed. The mean age was 48.5 years (SD=14.3; range=18-87) and the average MSE was 0.08 D (SD=2.14). Association testing was conducted on 2276701 SNPs which passed our quality control filters. The mean minor allele frequency was 0.X (SD=0.X), the average genotyping call rate was X% and none of the SNPs departed significantly from HWE. Counts of cases and controls and genomic control values are given in Table 1 of the paper. No SNPs achieved genome-wide significance in this sample (Supplementary Figure 6).

***Rotterdam Eye Studies 1, 2 and 3***

5238 individuals from RS1, 2009 individuals from RS2 and 1970 individuals from RS3 were analysed. The mean ages were 68.5 (SD=8.6, range=55-99) for RS1, 64.2 (SD=7.4, range=55-95) for RS2 and 60.8 (SD=5.5, range=45-86) for RS3. Association testing was conducted on S SNPs which passed our quality control filters. The mean minor allele frequency was NNN (SD=N), the average genotyping call rate was X% and none of the SNPs departed significantly from HWE. Counts of cases and controls and genomic control values are given in Table 1 of the paper. No SNPs achieved genome-wide significance in this sample (Supplementary Figures 7, 8 and 9).

***Meta-Analysis of the Replication Cohorts***

None of the selected replication SNPs achieved our replication thresholds for myopia or hyperopia (Supplementary Tables S6 and S7).
