# Supplementary material for: Genome-Wide Meta-Analysis of Myopia and Hyperopia Provides Evidence for Replication of 11 Loci
Source: PLoS One. 2014 Sep 18;9(9):e107110. doi: 10.1371/journal.pone.0107110 (PMC4169415; doi:10.1371/journal.pone.0107110)
Supplement: Materials S3 — Supplementary References. (DOCX) [file pone.0107110.s022.docx]

**SUPPLEMENTAL MATERIAL S3 – SUPPLEMENTAL REFERENCES**

1 Rahi, J.S., Cumberland, P.M. and Peckham, C.S. (2011) Myopia over the lifecourse: prevalence and early life influences in the 1958 British birth cohort. *Ophthalmology*, **118**, 797-804.

2 Vitart, V., Bencic, G., Hayward, C., Herman, J.S., Huffman, J., Campbell, S., Bucan, K., Zgaga, L., Kolcic, I., Polasek, O. *et al.* (2010) Heritabilities of ocular biometrical traits in two croatian isolates with extended pedigrees. *Invest Ophthalmol Vis Sci*, **51**, 737-743.

3 Vitart, V., Bencic, G., Hayward, C., Skunca Herman, J., Huffman, J., Campbell, S., Bucan, K., Navarro, P., Gunjaca, G., Marin, J. *et al.* (2010) New loci associated with central cornea thickness include COL5A1, AKAP13 and AVGR8. *Hum Mol Genet*, **19**, 4304-4311.

4 Spector, T.D. and Williams, F.M. (2006) The UK Adult Twin Registry (TwinsUK). *Twin Res Hum Genet*, **9**, 899-906.

5 Klein, R., Lee, K.E., Gangnon, R.E. and Klein, B.E. (2010) The 25-year incidence of visual impairment in type 1 diabetes mellitus the wisconsin epidemiologic study of diabetic retinopathy. *Ophthalmology*, **117**, 63-70.
